# Studies on the impact of modifications at the Gln-Trp site in RM2-based GRPR ligands

**DOI:** 10.1186/s13550-025-01241-7

**Published:** 2025-09-01

**Authors:** Sebastian Fischer, Lena Koller, Sandra Dominelli, Roswitha Beck, Hans-Jürgen Wester, Thomas Günther

**Affiliations:** 1 TUM School of Natural Sciences, Department of Chemistry, Chair of Pharmaceutical Radiochemistry, Technical University of Munich, 85748 Garching, Germany; 2 Department of Radiology, School of Medicine, Molecular Imaging Program at Stanford (MIPS), Stanford University, Stanford, CA 94305 USA

**Keywords:** RM2, Modified pharmacophore, GRPR, Antagonist, Metabolic stability

## Abstract

**Background:**

One of the most studied, and preclinically as well as clinically applied gastrin-releasing peptide receptor (GRPR) ligands represents the antagonist RM2 (DOTA-Pip^5^-D-Phe^6^-Gln^7^-Trp^8^-Ala^9^-Val^10^-Gly^11^-His^12^-Sta^13^-Leu^14^-NH_2_). As an improved in vivo stability was observed for a RM2 analog comprising the unnatural amino acid *α*-methyl-L-tryptophan instead of L-Trp, we aimed to elucidate the impact of other unnatural amino acids (homoserine [Hse], *β*-(3-benzothienyl)alanine [Bta]) at the metabolically less stable Gln-Trp site. Furthermore, we conjugated either DOTA, NOTA or NODAGA to the RM2 peptide and its modified derivatives, and evaluated each analog preclinically using ^68^Ga and ^64^Cu, as well as ^177^Lu (only DOTA-comprising compounds).

**Results:**

GRPR affinity and lipophilicity of RM2 derivatives were in a range of 1.2–8.4 nM and − 2.9 to − 1.1 (^nat/68^Ga-labeled), 1.7–33.0 nM and − 2.4 to − 1.6 (^nat/64^Cu-labeled), as well as 3.0–19.7 nM and − 3.2 to − 1.8 (^nat/177^Lu-labeled), respectively. Both, [^177^Lu]Lu-[Hse^7^]RM2 and [^177^Lu]Lu-[Bta^8^]RM2 revealed distinctly lower in vivo stability (< 20% intact at 15 min post-injection) than [^177^Lu]Lu-[*α*-Me-Trp^8^]RM2 (= [^177^Lu]Lu-AMTG) and [^177^Lu]Lu-RM2 (> 30% intact at 30 min post-injection). Both [^68^Ga]Ga-RM2 and [^68^Ga]Ga-AMTG exhibited high tumor (~ 15 percentage injected dose per gram, %ID/g) and pancreas uptake (> 25%ID/g), whereas [^68^Ga]Ga-[Hse^7^]RM2 and [^68^Ga]Ga-[Bta^8^]RM2 revealed lower tumor (~ 7.5%ID/g) but also substantially lower pancreas uptake (< 8%ID/g) at 1 h post-injection. At 24 h post-injection (p.i.), [^177^Lu]Lu-RM2 and [^177^Lu]Lu-AMTG exhibited high (> 8% ID/g) while [^177^Lu]Lu-[Hse^7^]RM2 and [^177^Lu]Lu-[Bta^8^]RM2 displayed low tumor retention (~ 2%ID/g). All compounds showed low activity levels in the pancreas at 24 h post-injection (< 1%ID/g).

**Conclusion:**

Substitution of the Gln-Trp site in RM2 by artificial amino acids had a distinct impact on overall pharmacokinetics. While Hse (instead of Gln) and Bta (instead of Trp) led to a decreased, *α*-Me-Trp (instead of Trp) led to an increased in vivo stability, which resulted in improved pharmacokinetics over time in case of the latter. However, at 1 h post-injection both [^68^Ga]Ga-[Hse^7^]RM2 and [^68^Ga]Ga-[Bta^8^]RM2 displayed slightly higher tumor-to-pancreas and tumor-to-intestine ratios, rendering homoserine and *β*-(3-benzothienyl)alanine potential options for the modification of GRPR ligands with regard to imaging properties.

**Supplementary Information:**

The online version contains supplementary material available at 10.1186/s13550-025-01241-7.

## Background

Driven by the ascension of the field of nuclear medicine over the last years due to attractive targets like the prostate-specific membrane antigen (PSMA), somatostatin-2 receptor (SST2R), and the fibroblast activation protein-*α* (FAP), preclinical research is facing the demand of imaging and treatment for a variety of further malignancies and their subtypes, and is thus encouraged to discover novel promising targets and develop respective tracers [1]. One well-studied target leading to the development and clinical translation of several high-affinity compounds [2] represents the gastrin-releasing peptide receptor (GRPR, bombesin-2 receptor).

Most work on GRPR-targeted compounds has been done in prostate cancer (PCa), as GRPR is overexpressed in the majority of PCa lesions [3], particularly in early, androgen-dependent stages of PCa [4], but also in late-stage neuroendocrine PCa [5], which could be addressing tumors with absent or low PSMA expression. Additionally, GRPR expression was observed in the majority of breast cancer (BCa), especially when estrogen receptor was also expressed [6]. Recent reports also showed a promising use of GRPR ligands for imaging of gastrointestinal stromal tumors [7, 8], rendering radiolabeled GRPR-targeted compounds promising for theranostic applications in different cancers. As adverse effects (nausea, diarrhea or abdominal cramps) were observed after administration of GRPR agonists into patients [9], GRPR antagonists such as RM2 (DOTA-Pip^5^-D-Phe^6^-Gln^7^-Trp^8^-Ala^9^-Val^10^-Gly^11^-His^12^-Sta^13^-Leu^14^-NH_2_) were preferred over the last decade [2].

While several radiolabeled GRPR ligands have been successfully used for positron emission tomography/computed tomography (PET/CT) imaging, the limited in vivo stability observed for linear GRPR-targeted peptides [10, 11] has restricted their use for treatment. We recently reported the stabilization of RM2 analogs by the introduction of an *α*-methyl-L-tryptophan moiety (instead of L-tryptophan), which led to a significantly higher in vivo stability and thus, improved overall pharmacokinetics preclinically and clinically [12–14]. This small modification addresses a major cleavage site (Gln^7^-Trp^8^) ubiquitously present in linear bombesin-based compounds, and hampers enzymatic cleavage by neprilysine (neutral endopeptidase, EC 3.4.24.11) [15, 16]. As endogenous enzymes are specialized to recognize natural amino acids, we assume that substitution of natural by unnatural amino acids in GRPR ligands can improve their in vivo stability, which has also been observed for other GRPR-targeted compounds [17, 18].

While lutetium-177 (^177^Lu) represents the most commonly used radionuclide in nuclear medicine for treatment, the most often applied radionuclides for PET imaging are fluorine-18 (^18^F) due to its attractive physical properties that allow for a high image resolution, and gallium-68 (^68^Ga) because of its good availability and simple labeling chemistry with the chelator 1,4,7,10-tetraazacyclododecan-1,4,7,10-tetracetic acid (DOTA). Another PET isotope that showed promising results in recent clinical studies represents copper-64 (^64^Cu). Despite its unfavorably low positron abundance of approximately 18%, it contains a similarly low positron energy to ^18^F (*E*_β+,max_: 653 keV versus 635 keV), which allows for high resolution PET imaging [19]. In addition, the substantially longer half-life of ^64^Cu compared with ^18^F and ^68^Ga (*t*_1/2_ = 12.7 h versus 109.7 min and 68 min, respectively) enables additional imaging at later time points, which proved to be beneficial for the detection of metastases with a low target expression [20].

Although [^64^Cu]Cu-DOTATATE has successfully been applied for imaging of neuroendocrine tumors [21], DOTA does not represent an ideal chelator for copper. As copper prefers a coordination number of six but DOTA (assuming that one carboxylic arm is used for the conjugation of the peptide) offers seven donors (four nitrogen atoms and three carboxylic arms), the resulting ^64^Cu-DOTA chelate is unstable in vivo, and thus leads to a steady release of the radiometal over time, particularly visible by elevated liver uptake [22–24]. Although ^68^Ga-DOTA chelates have demonstrated sufficient stability in vivo despite also preferring a coordination number of six, other chelators such as 1,4,7-triazacyclononane-1,4,7-triacetic acid (NOTA) or 1,4,7-triazacyclononane,1-glutaric acid-4,7-acetic acid (NODAGA) might be more suitable to form a stable complex with ^64^Cu or ^68^Ga. The radiometal-chelate can have a significant impact on in vitro and in vivo properties of the radiopharmaceutical, which was reported for RM26 derivatives [25, 26].

**Fig. 1:**
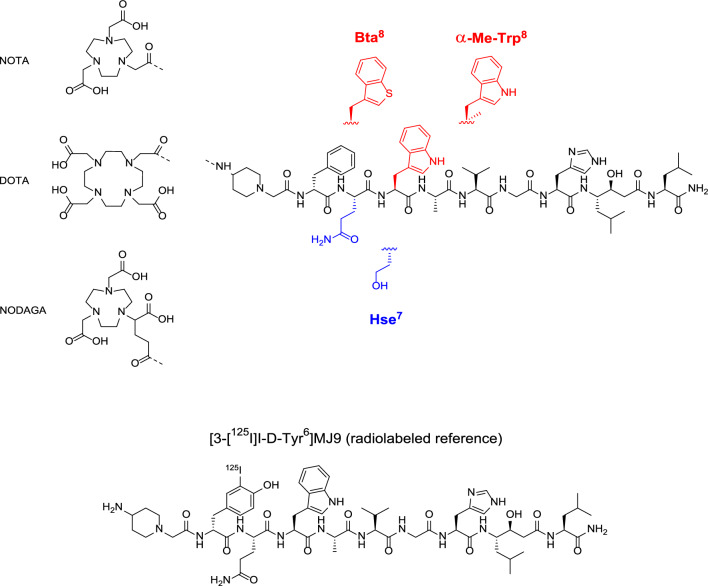
Chemical structure of the parent compound RM2 (black) including the DOTA chelator and its NOTA and NODAGA analogs, as well as the structural modifications at position 7 (homoserine [Hse], highlighted in blue) and 8 (*β*-(3-benzothienyl)alanine [Bta^8^], *α*-methyl tryptophan [*α*-Me-Trp^8^], highlighted in red), and chemical structure of the radiolabeled reference, [3-[^125^I]I-D-Tyr^6^]MJ9, used for all *IC*_50_ studies

With the aim to further evaluate the impact of modifications in linear GRPR-targeted peptides, we substituted the potent GRPR antagonist RM2 at the crucial Gln^7^-Trp^8^ site either by the unnatural amino acid homoserine (Hse) at position 7, or by *β*-(3-benzothienyl)alanine (Bta) or *α*-methyl tryptophan (*α*-Me-Trp) at position 8. Furthermore, to investigate the impact of the chelator and the radiometal on in vitro and in vivo data, each analog was synthesized either with DOTA, NOTA, or NODAGA attached (Fig. 1). All compounds were evaluated preclinically using the radionuclides gallium-68 (in vitro and in vivo) and copper-64 (in vitro), whereas lutetium-177 was only used for DOTA-comprising analogs (in vitro and in vivo).

## Materials and methods

Detailed description of the synthesis, labeling and characterization of RM2 and its analogs is provided in the supplementary materials.

The radiolabeled reference [3-[^125^I]I-D-Tyr^6^]MJ9 (Fig. 1) used for all *IC*_50_ studies was prepared by means of the Iodo-Gen® method according to a reported procedure [27].

### Chemical synthesis and labeling procedures

RM2 derivatives were prepared via standard Fmoc-based solid-phase peptide synthesis (SPPS) using a *H*-Rink amide ChemMatrix® resin (35–100 mesh particle size, 0.4–0.6 mmol/g loading, Merck KGaA, Darmstadt, Germany). Purification was accomplished by reversed-phase high-performance liquid chromatography (RP-HPLC). Electrospray ionization-mass spectra for characterization of the compounds were acquired on an expression^L^ CMS mass spectrometer (Advion Ltd., Harlow, UK). Labeling with [Cu/^64^Cu]copper, [Ga/^68^Ga]gallium, and [Lu/^177^Lu]lutetium was completed according to established procedures [12, 24]. [^64^Cu]CuCl_2_ was purchased from DSD-Pharma GmbH (Purkersdorf, Austria). Both[^68^Ga]GaCl_3_ and [^177^Lu]LuCl_3_ were acquired from ITM Isotope Technologies Munich SE (Garching, Germany).

### In Vitro* experiments*

*IC*_50_ values of all compounds were determined according to a previously published procedure [12]. Detailed information on culturing PC-3 cells and on the execution of *IC*_50_ studies is provided in the supplementary materials. In brief, competitive binding studies were performed in triplicate on PC-3 cells (1.5 × 10^5^ cells in 1 mL/well) after incubation at room temperature for 2 h, using [3-[^125^I]I-D-Tyr^6^]MJ9 (0.2 nM/well) as radiolabeled reference (n = 3).

Lipophilicity (depicted as *n*-octanol-phosphate-buffered saline solution (PBS, *pH* = 7.4) distribution coefficient, log*D*_7.4_) was determined by a standard protocol within our group [28]. Briefly, approximately 1 MBq of the labeled tracer was dissolved in 1 mL of a 1:1 mixture (v/v) of PBS (*pH* = 7.4) and *n*-octanol. After vigorous mixing for 3 min at room temperature and subsequent centrifugation (9,000 rpm, 5 min), 200 μL aliquots of both layers were measured in a *γ*-counter (Perkin Elmer Inc., Waltham, MA, USA). The experiment was repeated at least five times.

Metabolic stability in vitro was determined applying a procedure published by Linder et al*.* that was slightly modified [29]. Immediately after labeling, human serum (200 µL) was added to the labeling solution (~ 30 µL) and the mixture was incubated at 37 °C for 72 ± 2 h. Proteins were precipitated by treatment with ice-cold ethanol (150 µL) and ice-cold acetonitrile (450 µL), followed by centrifugation at 13,000 rpm for 5 min. The supernatants were decanted and further analyzed using radio RP-HPLC.

### In vivo experiments

Animal experiments were carried out according to the general animal welfare regulations in Germany (German animal protection act, in the edition of the announcement, dated 18 May 2006, as amended by Article 280 of 19 June 2020, approval no. ROB-55.2–1-2532.Vet_02-18–109 by the General Administration of Upper Bavaria) and the institutional guidelines for the care and use of animals. Female CB17-SCID mice aged 2–3 months (Charles River Laboratories International Inc., Sulzfeld, Germany) were used. After arrival at the in-house facilities, mice were allowed to acclimate for a minimum of one week before inoculation of PC-3 cells according to a previously reported protocol [12]. Animals would have been excluded from the study when reaching one of the following endpoints: a weight loss higher than 20%, a tumor size above 1,500 mm^3^, an ulceration of the tumor, respiratory distress or change of behavior. None of these criteria applied to any animal from the experiment. Neither randomization nor blinding was applied in the allocation of the experiments. Health status of the animals is specific pathogen free according to Federation of European Laboratory Animal Science Associations recommendation.

Metabolic stability in vivo was determined according to a previously published protocol [12] by injecting approximately 30–40 MBq (1 nmol, 150 µL) of the ^177^Lu-labeled compounds into the tail vein of anesthetized CB17-SCID mice (n = 3).

To establish tumor xenografts, PC-3 cells (5.0 × 10^6^ cells per 200 µL) were suspended in a 1/1 mixture (*v*/*v*) of Dulbecco’s modified eagle's medium/Ham’s F-12 with Glutamax-I (v/v = 1/1) and Cultrex® Basement Membrane Matrix Type 3 (Trevigen Inc., Gaithersburg, MD, USA) and inoculated subcutaneously onto the right shoulder of CB17-SCID mice (Charles River Laboratories International Inc., Sulzfeld, Germany). Mice (n = 4) were used for experiments when tumor volume has reached 125–500 mm^3^ (2–3 weeks after inoculation).

*µPET/CT imaging*. Imaging studies were performed at a MILabs VECTor^4^ small-animal SPECT/PET/OI/CT device (MILabs, Utrecht, the Netherlands). Data were reconstructed using the MILabs Rec software (version 10.02) and a pixel-based Similarity-Regulated Ordered Subsets Expectation Maximization (SROSEM) algorithm, followed by data analysis using the PMOD4.0 software (PMOD TECHNOLOGIES LLC, Zurich, Switzerland). For PET studies mice were anesthetized with 2% isoflurane, and ~ 2 MBq (100 pmol) of the ^68^Ga-labeled tracer were injected into the tail vein. Static images were recorded at 1 h p.i. with an acquisition time of 45–60 min (3–4 frames, 15 min each) using the HE-GP-RM collimator and a step-wise multi-planar bed movement. Depending on the size of the mouse, either four or eight bed position were required, with an imaging time of ~ 1.8 min or ~ 3.6 min per bed position. For competition studies, 3.62 mg/kg (40 nmol) of Lu-RM2 (10^−3^ M in PBS) were co-administered.

*Biodistribution.* Approximately 1–5 MBq (100 pmol) of the ^68^Ga- or ^177^Lu-labeled RM2 derivatives were injected into the tail vein of PC-3 tumor-bearing mice, and animals were sacrificed at 1 and 24 h p.i. (n = 4), respectively. Selected organs were removed, weighted and measured in a *γ*-counter.

Acquired data were statistically analyzed by performing either a one-way or two-way analysis of variance (ANOVA) test and a Tukey’s multiple comparisons test using the GraphPad Prism 10 software (GraphPad Software Inc*.*, San Diego, CA, USA). Acquired *p* values of < 0.05 were considered statistically significant.

## Results

### Chemical synthesis and radiolabeling

Synthesis via SPPS and subsequent RP-HPLC purification yielded 1-3% off-white solid (chemical purity > 95%, determined by RP-HPLC at λ = 220 nm). Complexation of all RM2 analogs (90 °C for 15 min) with a 2.5-fold excess of either Cu(OAc)_2_, Ga(NO_3_)_3_ or LuCl_3_ resulted in quantitative yields, and no further purification was conducted prior to *IC*_50_ studies. ^125^I-Iodination of [D-Tyr^6^]MJ9 (to obtain the radiolabeled reference for *IC*_50_ studies, [3-[^125^I]I-D-Tyr^6^]MJ9) by means of the Iodo-Gen® method resulted in radiochemical yields of 25% and radiochemical purities of more than 98% after RP-HPLC purification. In order to prevent radiolysis 10 vol-% of ascorbic acid (0.1 M in H_2_O) were added after purification.

Manual ^64^Cu-, ^68^Ga-, and ^177^Lu-Labeling of the respective compounds resulted in quantitative radiochemical yields, radiochemical purities of more than 95% and molar activities of 20 ± 1 (^64^Cu), 29 ± 5 (^68^Ga, decay corrected), and 38 ± 2 (^177^Lu) GBq/μmol, respectively.

### In Vitro* evaluation*

Affinity and lipophilicity data of all GRPR ligands examined in this study are summarized in Table 1.

**Table 1:** In vitro data of the RM2 derivatives comprising either DOTA, NOTA, or NODAGA as a chelator. Data are expressed as mean ± SD. Affinities of Ga-, Cu-, or Lu-complexed RM2 derivatives were determined on PC-3 cells (1.5 × 10^5^ cells/well) using [3-[^125^I]I-D-Tyr^6^]MJ9 (c = 0.2 nM) as radiolabeled reference (2 h, rt, HBSS + 1% BSA, *v*/*v*). Lipophilicity of ^68^Ga-, ^64^Cu-, and ^177^Lu-labeled GRPR ligands depicted as distribution coefficients at pH 7.4 (log*D*_7.4_). n.d. = not determined; ^#^ data taken from Koller et al. [24], these data have been determined in our lab under identical conditions; ^177^Lu-data taken from Guenther et al. [12], these data have been determined in our lab under identical conditions

GRPR ligand	*IC*_50_ (nM), Ga-labeled, n = 3	*IC*_50_ (nM), Cu-labeled, n = 3	*IC*_50_ (nM), Lu-labeled, n = 3	log*D*_7.4_, ^68^Ga- labeled, n = 6	log*D*_7.4_, ^64^Cu- labeled, n = 6	log*D*_7.4_, ^177^Lu-labeled, n = 6
RM2	2.1 ± 0.3^#^	3.2 ± 0.6^#^	3.5 ± 0.2	− 2.78 ± 0.03^#^	− 2.37 ± 0.04^#^	− 2.51 ± 0.02
[Hse^7^]RM2	7.7 ± 0.7	33.0 ± 2.7	19.7 ± 1.6	− 2.90 ± 0.09	− 2.04 ± 0.05	− 2.25 ± 0.06
[Bta^8^]RM2	2.3 ± 0.1	4.2 ± 0.6	4.6 ± 0.2	− 2.32 ± 0.07	− 1.73 ± 0.06	− 1.81 ± 0.02
AMTG	1.5 ± 0.2^#^	4.0 ± 0.5^#^	3.0 ± 0.0	− 2.26 ± 0.08^#^	− 2.17 ± 0.04^#^	− 2.28 ± 0.06
NOTA-[des-DOTA]RM2	1.2 ± 0.1	1.7 ± 0.3	n.d	− 1.93 ± 0.04	− 2.26 ± 0.02	n.d
NOTA-[des-DOTA, Hse^7^]RM2	8.4 ± 1.0	14.2 ± 2.7	n.d	− 1.56 ± 0.04	− 1.95 ± 0.04	n.d
NOTA-[des-DOTA, Bta^8^]RM2	1.8 ± 0.0	2.8 ± 0.2	n.d	− 1.28 ± 0.05	− 1.59 ± 0.05	n.d
NOTA-[des-DOTA, α-Me-Trp^8^]RM2	5.0 ± 0.7	5.1 ± 0.3	n.d	− 1.41 ± 0.02	− 1.79 ± 0.04	n.d
NODAGA-[des-DOTA]RM2	1.4 ± 0.2	3.1 ± 0.4	n.d	− 1.68 ± 0.03	− 2.14 ± 0.10	n.d
NODAGA-[des-DOTA, Hse^7^]RM2	7.2 ± 0.7	11.8 ± 2.1	n.d	− 1.51 ± 0.04	− 2.00 ± 0.04	n.d
NODAGA-[des-DOTA, Bta^8^]RM2	2.9 ± 0.6	8.9 ± 0.3	n.d	− 1.14 ± 0.03	− 1.62 ± 0.04	n.d
NODAGA-[des-DOTA, α-Me-Trp^8^]RM2	4.9 ± 0.7	12.6 ± 0.4	n.d	− 1.42 ± 0.04	− 1.94 ± 0.05	n.d

**Fig. 2:**
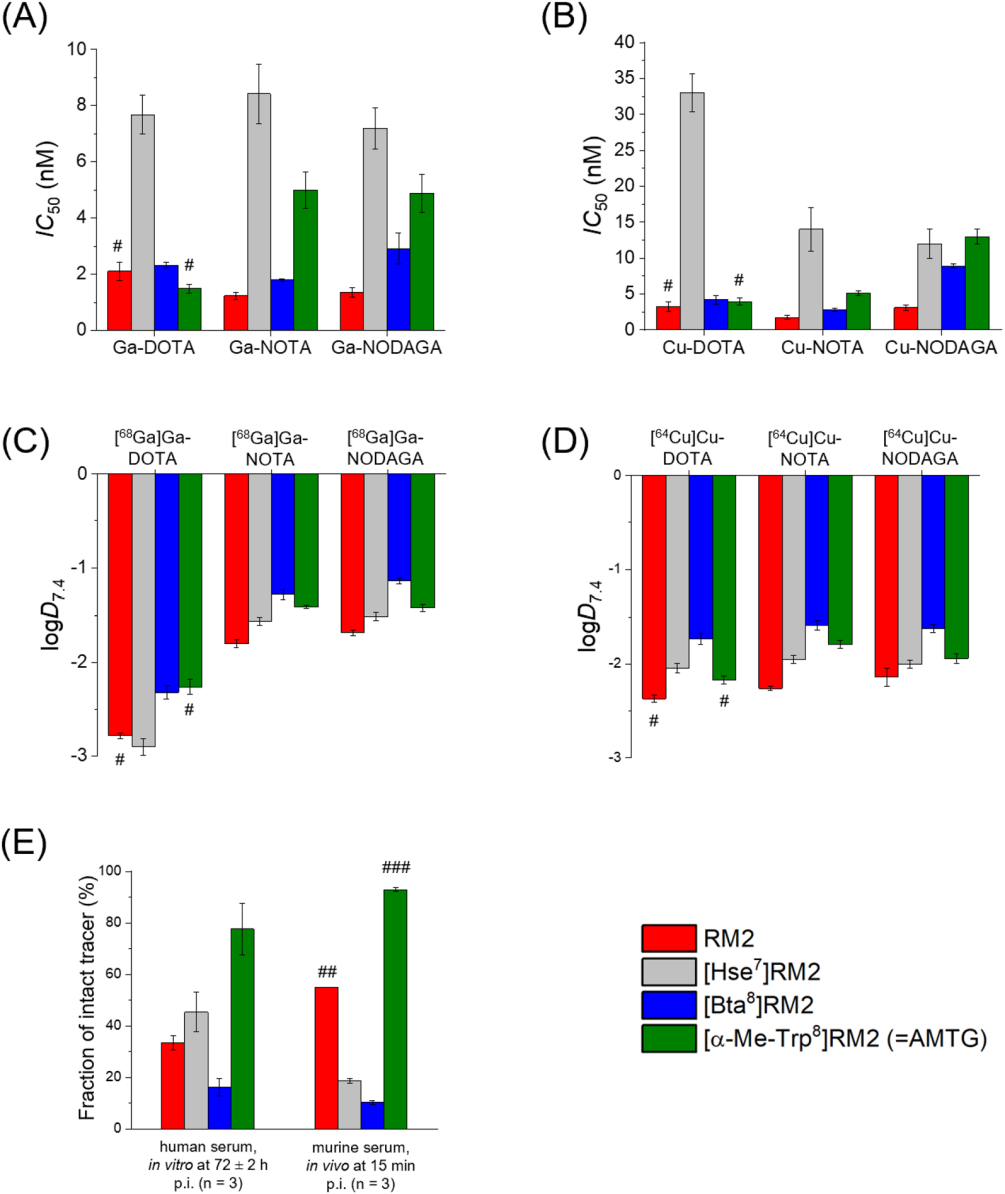
GRPR affinity (n = 3) and lipophilicity (n = 6) of either ^nat/68^Ga- or ^nat/64^Cu-complexed GRPR ligands containing either a DOTA, NOTA, or NODAGA chelator. *IC*_50_ values of **A** Ga- or **B** Cu-complexed GRPR ligands comprising different chelators. log*D*_7.4_ values of **C** ^68^Ga- or **D** ^64^Cu-complexed GRPR ligands using different chelators. **E** In vitro stability of [^177^Lu]Lu-DOTA-analogs after incubation in human serum at 37 °C for 72 ± 2 h (n = 3), and in vivo stability in murine organism at 15 min after injection. ^#^ data taken from Koller et al. [24], these data have been determined in our lab under identical conditions; ^##^ [^68^Ga]Ga-RM2, data taken from Popp et al. [30], these data have been determined by a different group using a similar protocol; ^###^ stability at 30 min after injection, data taken from Guenther et al. [12], these data have been determined in our lab under identical conditions; statistical information can be found in Figs. S1-2

*IC*_50_ values of Ga-labeled GRPR-targeted compounds were in a range of 1.2–8.4 nM (Fig. 2A), while their Cu-labeled analogs revealed *IC*_50_ values of 1.7–33.0 nM (Fig. 2B).

While the [*α*-Me-Trp^8^]RM2 (= AMTG) derivative containing a DOTA chelator revealed the highest GRPR affinity, irrespective of the metal incorporated, most of the other analogs demonstrated highest GRPR affinity when a NOTA chelator was conjugated. Noteworthy, the conjugated chelator had no significant impact on RM2, [Hse^7^]RM2, or [Bta^8^]RM2 analogs with regard to GRPR affinity (*p* > 0.05), whereas the AMTG derivatives showed a significant difference in the chelators attached (*p* < 0.0001) (**Fig. S2**). In general, lipophilicity was lowest for the [^68^Ga]Ga-DOTA-conjugated derivatives (log*D*_7.4_: − 2.9 to − 2.3), and highest for the [^68^Ga]Ga-NOTA- and the [^68^Ga]Ga-NODAGA-comprising compounds (log*D*_7.4_: − 1.9 to − 1.1, Fig. 2C *p*< 0.0001). In case of the ^64^Cu-labeled derivatives, no general preference for any chelator could be observed with regard to lipophilicity (log*D*_7.4_: − 2.4 to − 1.7 ([^64^Cu]Cu-DOTA), − 2.3 to − 1.6 ([^64^Cu]Cu-NOTA), − 2.1 to − 1.6 ([^64^Cu]Cu-NODAGA), Fig. 2D), as most differences were statistically significant but not as prominent as for the ^68^Ga-labeled analogs.

In vitro stability studies in human serum (incubation at 37 °C for 72 ± 2 h) using the ^177^Lu-labeled DOTA-conjugated derivatives revealed a substantially lower stability for all compounds (16–45% intact) when compared with [^177^Lu]Lu-AMTG (78% intact, Figs. S3A-D, *p* < 0.01).

### In vivo* evaluation*

Investigation of in vivo stability in healthy mice did not show any intact [^177^Lu]Lu-[Hse^7^]RM2 or [^177^Lu]Lu-[Bta^8^]RM2 present in the serum at 30 min after injection, while 33% and 92% of [^177^Lu]Lu-RM2 and [^177^Lu]Lu-AMTG, respectively, were still intact at the same time point. At 15 min after injection ~ 19% ([^177^Lu]Lu-[Hse^7^]RM2) and ~ 10% ([^177^Lu]Lu-[Bta^8^]RM2) were intact (Fig. 2E, Figs. S3E-H) in the serum.

**Fig. 3:**
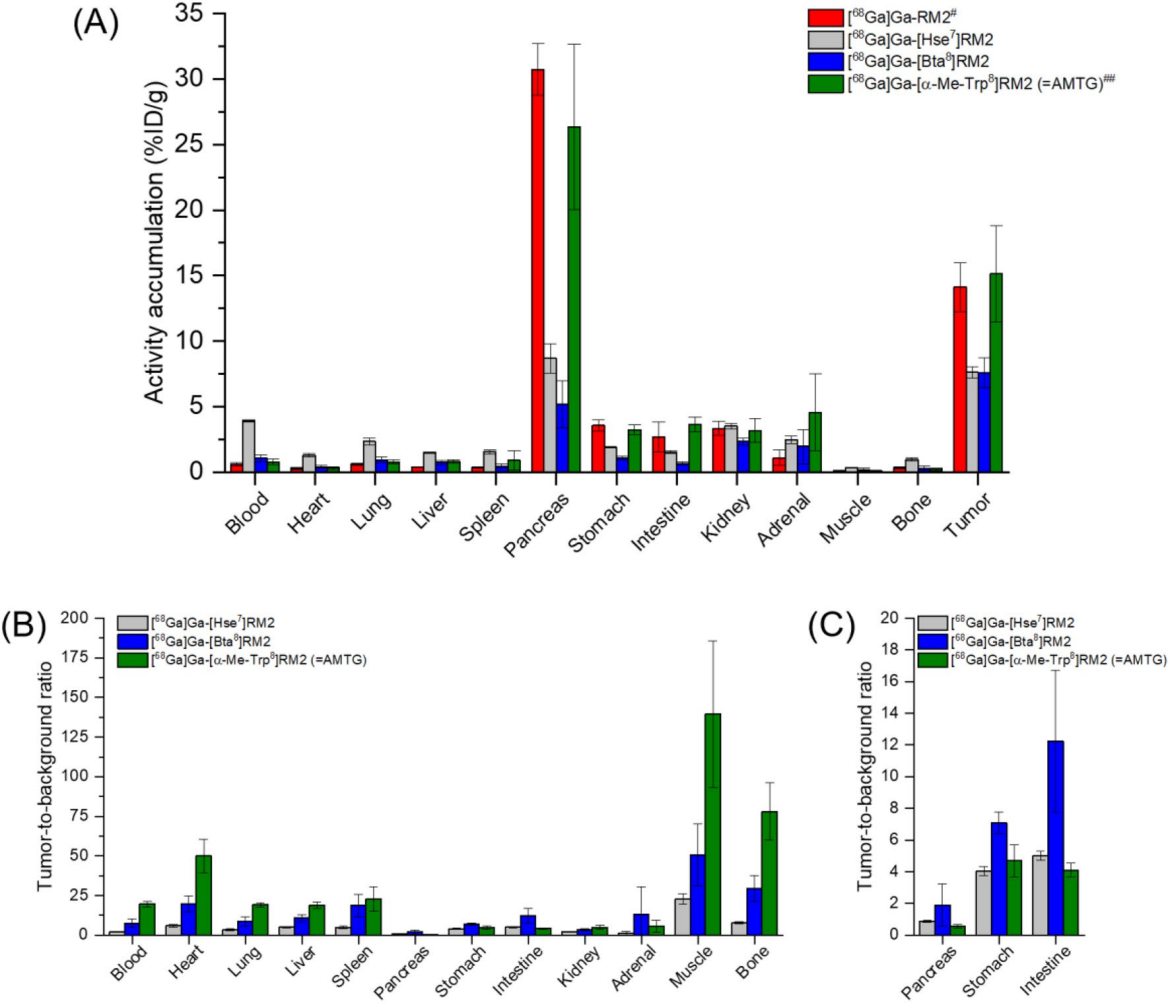
In vivo data of ^68^Ga-labeled RM2 derivatives in PC-3 tumor-bearing CB17 SCID mice at 1 h p.i. (n = 4 each, 100 pmol each). **A** Biodistribution and **B** tumor-to-background ratios, as well as **C** tumor-to-pancreas, tumor-to-stomach, and tumor-to-intestine ratios of ^68^Ga-labeled GRPR ligands. ^#^ data taken from Mansi et al. [31], these data have been determined by a different group using a similar protocol; ^##^ data taken from Koller et al. [24], these data have been determined in our lab under identical conditions; statistical information can be found in Fig. S4

Biodistribution profiles in PC-3 tumor-bearing mice were favorable for all ^68^Ga-labeled GRPR ligands at 1 h p.i. (Fig. 3A, Table S1).

**Fig. 4:**
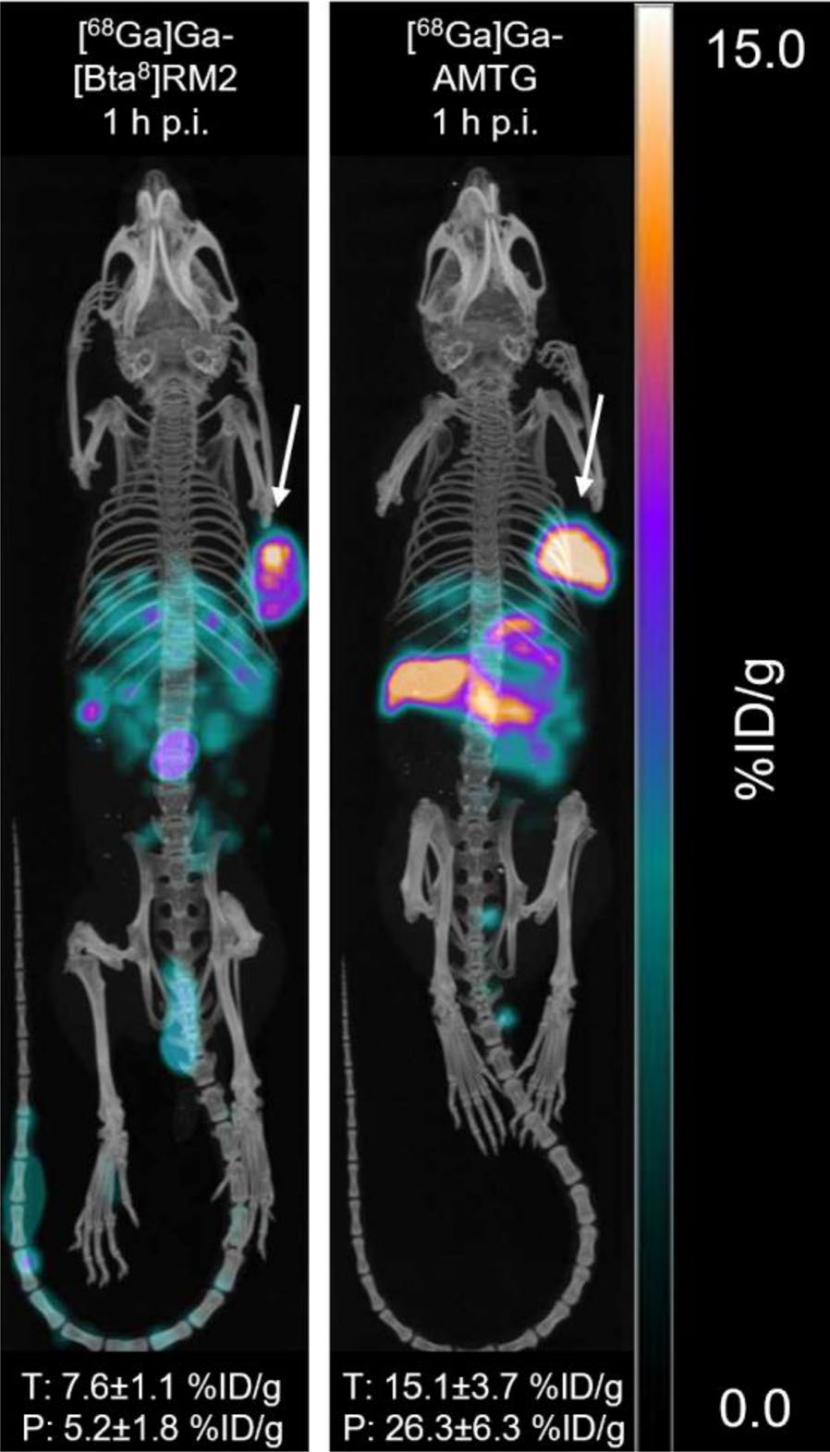
µPET/CT images of PC-3 tumor-bearing CB17 SCID mice 1 h after injection of either [^68^Ga]Ga-[Bta^8^]RM2 or [^68^Ga]Ga-AMTG (100 pmol each). White arrows illustrate the location of the tumor

While [^68^Ga]Ga-RM2 and [^68^Ga]Ga-AMTG displayed highest tumor uptake (~ 15 percentage injected dose per gram, %ID/g), [^68^Ga]Ga-[Bta^8^]RM2 and [^68^Ga]Ga-[Hse^7^]RM2 revealed significantly lower activity levels in the tumor xenograft (~ 7.5%ID/g, *p* < 0.001). However, both compounds also exhibited significantly lower accumulation in the pancreas than [^68^Ga]Ga-AMTG (~ 5–8 versus ~ 26%ID/g, *p* < 0.0001). Hence, whereas [^68^Ga]Ga-AMTG revealed highest tumor-to-background ratios in most organs, [^68^Ga]Ga-[Bta^8^]RM2 showed highest tumor-to-pancreas (*p* > 0.5), tumor-to-stomach (*p* > 0.1), and tumor-to-intestine (*p* < 0.001) ratios among all compounds tested (Figs. 3B-C, Table S2). µPET/CT imaging in PC-3 tumor-bearing mice (100 pmol each) confirmed low abdominal uptake of [^68^Ga]Ga-[Bta^8^]RM2 at 1 h p.i., while [^68^Ga]Ga-AMTG illustrated substantially higher activity accumulation in the tumor xenograft (Fig. 4).

**Fig. 5:**
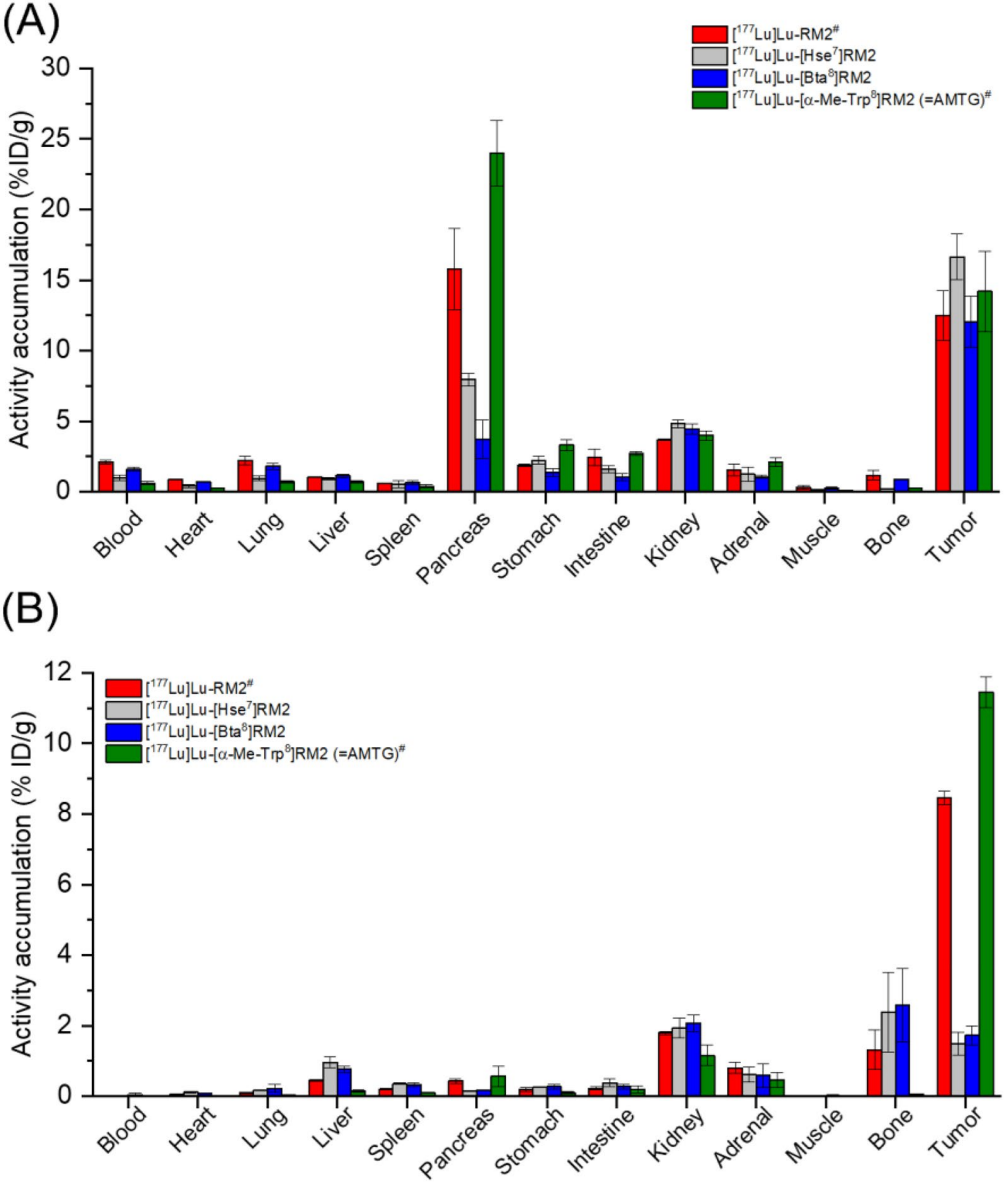
In vivo data of ^177^Lu-labeled RM2 derivatives in PC-3 tumor-bearing CB17 SCID mice at **A** 1 h p.i. and **B** 24 h p.i. (n = 4 each, 100 pmol each). ^#^ data taken from Holzleitner et al. [32], these data have been determined in our lab under identical conditions; statistical information can be found in Fig. S5

Biodistribution studies of the four ^177^Lu-labeled RM2 analogs evaluated revealed a similar overall profile to their ^68^Ga-labeled counterparts at 1 h p.i. (n = 4 each, 100 pmol each), displaying highest activity uptake in the tumor xenograft and the pancreas (Fig. 5A, Table S3).

However, while [^177^Lu]Lu-RM2 and [^177^Lu]Lu-AMTG demonstrated similarly high tumor uptake compared with their respective ^68^Ga-labeled analogs at 1 h p.i., both [^177^Lu]Lu-[Hse^7^]RM2 and [^177^Lu]Lu-[Bta^8^]RM2 showed substantially higher tumor accumulation than their ^68^Ga-labeled counterparts at 1 h p.i., whereas activity levels in the pancreas were similarly low. Nevertheless, while high activity levels of both [^177^Lu]Lu-RM2 and [^177^Lu]Lu-AMTG were retained in the tumor xenograft at 24 h p.i. (Fig. 5B), most of the activity of [^177^Lu]Lu-[Hse^7^]RM2 and [^177^Lu]Lu-[Bta^8^]RM2 observed in the tumor at 1 h p.i. was cleared at 24 h p.i. This resulted in low activity levels in the tumor xenograft at 24 h p.i. (n = 4 each, 100 pmol each, Table S4) and thus, substantially lower tumor-to-background ratios at 24 h p.i. in most organs (Fig. S6A). Tumor accumulation for both [^177^Lu]Lu-[Hse^7^]RM2 and [^177^Lu]Lu-[Bta^8^]RM2 was found to be specific, as competition with excess of Lu-RM2 exhibited noticeably reduced tumor uptake at 1 h p.i. (Fig. S6B).

## Discussion

In vivo stability of radiopharmaceuticals is one of the pivotal aspects of their therapeutic efficacy, as a higher stability is typically accompanied with a higher bioavailability in the blood pool and a longer retention at the target site. While small molecules or cyclic compounds usually display high in vivo stability, linear peptides like GRPR-targeted peptides are prone to enzymatic degradation. In previous studies we observed a major instability in the Gln^7^-Trp^8^ sequence, which is ubiquitously present in all bombesin-based compounds, and which is enzymatically tackled by neprilysine [16]. Based on the positive effect of an Trp-for-*α*-Me-Trp substitution, which led to a substantially improved in vivo stability preclinically [12, 32] and clinically [13], we aimed to further evaluate the impact of amino acid substitutions (Gln-for-Hse^7^ and Trp-for-Bta^8^ switch) in RM2-based peptides.

Synthesis of all compounds was accomplished via Fmoc-based solid-phase peptide synthesis and yielded 1-3% of unlabeled precursor after RP-HPLC purification without any optimization of the synthesis efficiency. ^nat/68^Ga- and ^nat/64^Cu-complexations of all GRPR ligands were quantitative using established protocols, irrespective of which chelator (DOTA, NOTA, or NODAGA) was conjugated. ^nat/177^Lu-Complexation of DOTA-comprising GRPR ligands was also quantitative when applying standard labeling procedures [28].

Despite the small structural variations between the four peptide sequences (RM2, [Hse^7^]RM2, [Bta^8^]RM2, AMTG), we observed substantial differences in GRPR affinity, lipophilicity, and in vivo stability. While RM2 revealed very high affinity irrespective of which chelator was conjugated and which metal was complexed, the [Hse^7^]RM2 derivatives displayed the lowest affinity. This points to an importance of the amide moiety of Gln for high affinity binding to the receptor, as substitution by a hydroxyl moiety (Hse) had a substantial impact. In general, the Hse modification led to a slightly higher lipophilicity compared with the unmodified RM2 analogs, assuming the polarity of a hydroxyl group is similar to that of an amide moiety. Affinity of the [Bta^8^]RM2 analogs was observed to be very close to that of the RM2 analogs (except when NODAGA was attached), which was expected as those compounds only differ in one atom (nitrogen versus sulfur atom in the indole moiety of Trp). However, this tiny difference had a significant impact on lipophilicity, as all Bta-comprising derivatives exhibited a noticeably higher lipophilicity than their respective RM2 analogs.

Interestingly, while the introduction of a methyl group at the *α*-carbon of the Trp moiety (AMTG derivatives) did not affect GRPR affinity when DOTA is conjugated as a chelator, AMTG derivatives containing NOTA or NODAGA revealed a significantly lower GRPR affinity compared with their RM2 counterparts. This indicates that the overall complexation geometry of the metal-chelate impacts the affinity of modified RM2 analogs differently. A similar observation was made for the Cu-labeled [Hse^7^]RM2 derivatives. Although the impact of the *α*-Me-Trp modification on lipophilicity was not as distinct as for the Bta modification, it was still surprising that the addition of a small methyl group to the different RM2 derivatives revealed a pronounced impact.

Worth mentioning, while the ^68^Ga-DOTA chelate revealed a substantially lower lipophilicity than the ^68^Ga-NOTA or the ^68^Ga-NODAGA, irrespective of the modification in the peptide sequence, the different ^64^Cu-chelates did not impact the lipophilicity of the respective compounds. This was unexpected, as both Ga^3+^ and Cu^2+^ comprise a coordination number of 6, which leads to an overall negatively charged chelate with DOTA because the four nitrogen atoms and two carboxyl groups form the complex, while the third carboxyl group remains free (forth carboxyl group of DOTA used for conjugation of the peptide). As both NOTA and NODAGA contain less nitrogen atoms than DOTA (3 versus 4) and a less (NOTA) or similar number (NODAGA) of carboxylic moieties, the overall charge of the Ga/Cu-NOTA or Ga/Cu-NODAGA chelate is positive and neutral, respectively [25]. Therefore, the lowest lipophilicity was anticipated for the negatively charged ^68^Ga/^64^Cu-DOTA chelate, irrespective of the modification present within the peptide sequence, which was observed for ^68^Ga but not for ^64^Cu. This surprising finding has to be investigated in future studies, and maybe also using other peptides to elucidate whether this is a general difference between the ^68^Ga- and the ^64^Cu-DOTA chelate.

In previous studies we already observed the beneficial effect of a substitution of Trp by *α*-Me-Trp in RM2, which resulted in substantially higher in vivo stability preclinically and clinically [12, 13, 32]. Although we anticipated that the substitution of natural (Gln^7^, Trp^8^) by unnatural (Hse^7^, Bta^8^, *α*-Me-Trp^8^) amino acids will lead to an improved metabolic stability as a consequence of a more difficult recognition of artificial amino acids by enzymes, we could not detect any intact [^177^Lu]Lu-[Hse^7^]RM2 or [^177^Lu]Lu-[Bta^8^]RM2 in the murine organism at 30 min after injection. Therefore, intact percentages of both compounds were investigated at 15 min after injection, which revealed that less than 20% of the injected [^177^Lu]Lu-[Hse^7^]RM2 and [^177^Lu]Lu-[Bta^8^]RM2, respectively, was still intact, while more than 90% of [^177^Lu]Lu-AMTG were still intact in the murine organism at 30 min after injection. Popp et al. reported that 55% of [^68^Ga]Ga-RM2 were still intact in the murine organism at 15 min after injection [30], indicating an unfavorable effect of both the Hse and Bta modification on in vivo stability.

A possible explanation for this observation could be that the enzyme mostly responsible for the cleavage of linear GRPR ligands, neprilysine, is known to cleave peptide sequences at the N-terminal side of hydrophobic sites i.e., Gln^7^-Trp^8^ [15, 16]. As particularly the Bta but also the Hse modification led to an increased overall lipophilicity, and these were the only difference compared with the unmodified RM2 peptide, we assume that the enhanced hydrophobic character generated by these modifications directly at the site most vulnerable towards enzymatic cleavage additionally triggered neprilysine activity. An exception thereto represents the *α*-Me-Trp modification, as its additional methyl group at the *α*-carbon likely hampers the docking of the compound to the active center of the enzyme, thus causing a higher protection from enzymatic cleavage. Noteworthy, [^177^Lu]Lu-[Hse^7^]RM2 displayed higher in vitro stability in human serum than [^177^Lu]Lu-RM2 after incubation at 37 °C for 72 ± 2 h, which contradicts the in vivo results observed in mice. Species differences between humans and mice are apparent, and thus could lead to opposite results. However, we assume that the difference in enzymatic activity in vitro and in vivo is more prominent [16], and thus likely leads to these contradicting observations. It has to be mentioned that we value stability results in vivo more than in vitro, as the former provides a more realistic scenario to a potential in vivo application in humans despite the species differences, as in vivo studies take into account the presence of enzymes not located in the blood serum, for example neprilysine, which is mainly expressed in kidney tissue.

In order to investigate the importance of an intact sequence for high GRPR affinity, we synthesized the suspected fragments that would result if our compounds were cleaved at position 7 or 8. None of the potential fragments demonstrated any GRPR affinity (Table S5), underlining the pivotal role of in vivo stability not only for retention but also for the accumulation of the drug at the target.

Based on the favorable overall in vitro data of the [^68^Ga]Ga-DOTA-containing RM2 derivatives we decided to compare these four compounds in vivo at 1 h after injection. Furthermore, we aimed to investigate a longer-term impact of the modifications on biodistribution, thus evaluating their [^177^Lu]Lu-DOTA-containing analogs at 24 h but also at 1 h after injection. Biodistribution studies at 1 h post-injection illustrated high tumor and pancreas uptake for the more stable [^68^Ga]Ga-RM2 and [^68^Ga]Ga-AMTG, while overall off-target uptake was low. In contrast, both [^68^Ga]Ga-[Hse^7^]RM2 and [^68^Ga]Ga-[Bta^8^]RM2 demonstrated significantly lower activity levels in the tumor xenograft (7.5%ID/g versus 14–15%ID/g) and the pancreas (5–9%ID/g versus 26–30%ID/g), which can be attributed to their lower in vivo stability and thus, accelerated activity clearance. Similar observations were made for [^68^Ga]Ga-TacBOMB2 (LW01085), which revealed poor in vivo stability and thus, low pancreas (1.30 ± 0.14%ID/g) and moderate tumor uptake (5.95 ± 0.50%ID/g) at 1 h p.i. [33]. However, it has to be mentioned that other compounds developed by the same group such as [^68^Ga]Ga-ProBOMB1, [^68^Ga]Ga-TacsBOMB2, [^68^Ga]Ga-TacsBOMB5, [^68^Ga]Ga-LW02002, and [^68^Ga]Ga-LW01142, among others, exhibited a higher percentage of intact compound than [^68^Ga]Ga-TacBOMB2 in murine plasma at 15 min after injection, but each of these compounds still revealed low activity levels in the pancreas at 1 h post-injection [17, 18, 34, 35], suggesting that clearance from the pancreas might not mainly be driven by metabolic stability. While off-target uptake was low for [^68^Ga]Ga-[Bta^8^]RM2, it was elevated for [^68^Ga]Ga-[Hse^7^]RM2 in blood, heart, lung, liver and spleen, indicating the Hse modification slightly increases albumin binding and thus results in a decelerated activity clearance. Although both compounds revealed a slightly higher tumor-to-pancreas (not significant) and tumor-to-intestine (statistically significant) ratio than [^68^Ga]Ga-AMTG, tumor-to-organ ratios were noticeably lower in all other organs.

Interestingly, while both [^68^Ga]Ga-RM2 and [^68^Ga]Ga-AMTG as well as their ^177^Lu-labeled analogs showed similar biodistribution profiles at 1 h post-injection, both [^177^Lu]Lu-[Hse^7^]RM2 and [^177^Lu]Lu-[Bta^8^]RM2 exhibited more favorable profiles than their ^68^Ga-labeled counterparts at 1 h after injection, which was in accordance to the data reported for [^68^Ga]Ga-/[^177^Lu]Lu-ProBOMB2 [36]. Overall activity uptake in non-tumor organs was low, even for [^177^Lu]Lu-[Hse^7^]RM2 although its ^68^Ga-labeled analog showed elevated uptake in many organs. Both [^177^Lu]Lu-[Hse^7^]RM2 and [^177^Lu]Lu-[Bta^8^]RM2 revealed high activity levels in the tumor xenograft at 1 h p.i., with the former even surpassing [^177^Lu]Lu-RM2 and [^177^Lu]Lu-AMTG. This was unexpected, as both [^177^Lu]Lu-[Hse^7^]RM2 and [^177^Lu]Lu-[Bta^8^]RM2 demonstrated lower GRPR affinity than their ^68^Ga-labeled analogs, as well as [^177^Lu]Lu-RM2 and [^177^Lu]Lu-AMTG. In order to evaluate whether this surprising, yet favorable result might be reflected at later time points as well, we carried out biodistribution studies at 24 h post-injection, which did not retain the favorable profiles observed at 1 h after injection, indicating that the poor in vivo stability of [^177^Lu]Lu-[Hse^7^]RM2 and [^177^Lu]Lu-[Bta^8^]RM2 limits their therapeutic value.

All three modifications led to substantially decreased GRPR affinity when used in the GRPR agonist LW01085 (TacBOMB2) [18], which indicates that even small modifications within a bombesin-based sequence can significantly impact its arrangement in the receptor, given the high structural similarity of GRPR antagonists and agonists, which mainly differ in only the two C-terminal positions. Similar observations were made for the substitution of N-Me-His (instead of His, data not shown), which led to noticeably decreased GRPR affinity in RM2 (*IC*_50_ ~ 1,500 nM) while several high-affinity TacBOMB2 derivatives that contain N-Me-His are reported [18]. Substitution of Trp by *α*-Me-Trp in the GRPR antagonist TacsBOMB2 (similar structure to the agonist TacBOMB2) did retain high GRPR affinity but did not show a similarly high benefit regarding in vivo stability and biodistribution [34].

We also introduced Hse, Bta, and *α*-Me-Trp in the clinically applied NeoB. Interestingly, the Bta modification led to the highest loss of GRPR affinity (Table S6). However, the impact of these modifications on in vitro stability in human serum showed the same trend as for our RM2 analogs. When we introduced the modifications in the GRPR agonist, UL (“Universal Ligand”, [D-Phe^6^, β-Ala^11^, Phe^13^, Nle^14^]Bn_6-14_), both Bta and *α*-Me-Trp led to a slightly decreased GRPR affinity, while Hse decreased affinity noticeably. Interestingly, both Hse and *α*-Me-Trp led to an improved stability in human serum, while Bta decreased stability. Taking all those observations into account, a general tool box of modifications that can be applied to optimize GRPR-targeted compounds does not seem to exist, and each GRPR antagonist (or agonist) has to be optimized individually.

In summary, we found that modifications at the crucial Gln^7^-Trp^8^ site in RM2 derivatives can have a significant impact on in vitro and in vivo properties. While substitution of Trp by *α*-Me-Trp (AMTG) led to a substantially improved in vivo stability, substitution of Gln by Hse or Trp by Bta resulted in noticeably decreased in vivo stability when compared with the parent compound RM2, which prevents their use as therapeutics. Despite a favorable effect on activity clearance from the pancreas and the intestine, which could result in improved imaging, both [^68^Ga]Ga-[Hse^7^]RM2 and [^68^Ga]Ga-[Bta^8^]RM2 suffered from limited uptake in the tumor xenograft, thus rendering both compounds inferior imaging characteristics. Interestingly, we have introduced both the Hse and the Bta modification in ^99m^Tc-labeled GRPR ligands, which revealed high tumor and low abdominal uptake at early time points in PC-3 tumor-bearing mice [37]. [^99m^Tc]Tc-N_4_-asp-[Bta^8^]MJ9 has already been translated into clinical practice, demonstrating a favorable biodistribution profile due to its rapid activity clearance [38]. Further studies on Hse/Bta-comprising GRPR ligands labeled with different radionuclides would be necessary in order to investigate possible fits for these modifications, as ^99m^Tc-labeled but not ^68^Ga-labeled bombesin analogs seem to be a good fit.

## Conclusion

Substitution of the Gln^7^-Trp^8^ sequence in the GRPR ligand RM2 exhibited insight into this sensitive site and showed which impact on in vitro and in vivo properties even small modifications can have. Although unnatural amino acids were introduced, a noticeably lower metabolic stability was found for homoserine or *β*-(3-benzothienyl)alanine modifications, which did not result in GRPR ligands suitable for a clinical use. Only [^177^Lu]Lu-AMTG (α-methyl-L-tryptophan modification) revealed a high in vivo stability over time and thus, shows the highest therapeutic value among radiolabeled GRPR ligands, which has also been confirmed in cancer patients. Regarding the chelators used, neither NOTA nor NODAGA demonstrated an advantage over DOTA in terms of GRPR affinity and lipophilicity.

## Supplementary Information

Supplementary file1

## Data Availability

Data is contained within the article and supplementary materials.

## Abbreviations

α-Me-Trp: *α*-Methyl tryptophan
BCa: Breast cancer
Bta: β-(3-Benzothienyl)alanine
CT: Computed tomography
DOTA: 1,4,7,10-Tetraazacyclododecan-1,4,7,10-tetracetic acid
FAP: Fibroblast activation protein-*α*
Fmoc: 9-Fluorenylmethoxycarbonyl
GRPR: Gastrin-releasing peptide receptor
Hse: Homoserine
IC_50_: Half-maximal inhibitory concentration
Iodo-Gen***®***: 1,3,4,6-Tetrachloro-3*R*,6*R*-diphenylglycoluril
logD_7.4_: Distribution coefficient (octanol/PBS) at pH = 7.4
MJ9: Pip-D-Phe-Gln-Trp-Ala-Val-Gly-His-Sta-Leu-NH_2_
n.d.: Not determined
NODAGA: 1,4,7-Triazacyclononane,1-glutaric acid-4,7-acetic acid
NOTA: 1,4,7-Triazacyclononane-1,4,7-triacetic acid
PBS: Phosphate-buffered saline solution
PCa: Prostate cancer
PET: Positron emission tomography
p.i.: Post-injection
PSMA: Prostate-specific membrane antigen
RM2: DOTA-Pip-D-Phe-Gln-Trp-Ala-Val-Gly-His-Sta-Leu-NH_2_
RP-HPLC: Reversed-phase high performance liquid chromatography
SST2R: Somatostatin-2 receptor
SPECT: Single-photon emission computed tomography
SPPS: Solid-phase peptide synthesis
v/v: Volume ratio

## References

[1] Zhang S, Wang X, Gao X, Chen X, Li L, Li G, et al. Radiopharmaceuticals and their applications in medicine. Signal Transduct Target Ther. 2025;10(1):1. 39747850 10.1038/s41392-024-02041-6 PMC11697352

[2] Zou Y, Huang M, Hu M, Wang H, Chen W, Tian R. Radiopharmaceuticals targeting gastrin-releasing peptide receptor for diagnosis and therapy of prostate cancer. Mol Pharm. 2024;21(9):4199–216. 39219355 10.1021/acs.molpharmaceut.4c00066

[3] Markwalder R, Reubi JC. Gastrin-releasing peptide receptors in the human prostate relation to neoplastic transformation. Cancer Res. 1999;59(5):1152–9. 10070977

[4] Schroeder RP, de Visser M, van Weerden WM, de Ridder CM, Reneman S, Melis M, et al. Androgen-regulated gastrin-releasing peptide receptor expression in androgen-dependent human prostate tumor xenografts. Int J Cancer. 2010;126(12):2826–34. 19876914 10.1002/ijc.25000

[5] Zhang H, Qi L, Cai Y, Gao X. Gastrin-releasing peptide receptor (GRPR) as a novel biomarker and therapeutic target in prostate cancer. Ann Med. 2024;56(1):2320301. 38442298 10.1080/07853890.2024.2320301 PMC10916925

[6] Morgat C, MacGrogan G, Brouste V, Velasco V, Sevenet N, Bonnefoi H, et al. Expression of gastrin-releasing peptide receptor in breast cancer and its association with pathologic, biologic, and clinical parameters: a study of 1,432 primary tumors. J Nucl Med. 2017;58(9):1401–7. 28280221 10.2967/jnumed.116.188011

[7] Gruber L, Decristoforo C, Uprimny C, Hohenberger P, Schoenberg SO, Orlandi F, Mariani MF, Manzl C, Kasseroler MT, Tilg H, Zelger B, Jaschke WR, Virgolini IJ. Imaging properties and tumor targeting of 68Ga-NeoBOMB1, a gastrin-releasing peptide receptor antagonist, in GIST patients. Biomedicines. 2022;10(11):2899. 10.3390/biomedicines10112899. 36428467 10.3390/biomedicines10112899 PMC9687401

[8] Wang R, Kang W, Liu Z, Zheng Y, Sui H, Li L, Wang J, Xiang J, Peng X, Chen X, Zhu Z, Zhang J. Head-to-head comparison of [68Ga]Ga-NOTA-RM26 and [18F]FDG PET/CT in patients with gastrointestinal stromal tumors: a prospective study. J Nuclear Med. 2024;66(2):201–6. 10.2967/jnumed.124.267810. 10.2967/jnumed.124.267810 39448271

[9] Mather SJ, Nock BA, Maina T, Gibson V, Ellison D, Murray I, et al. GRP receptor imaging of prostate cancer using [99mTc]Demobesin 4: a first-in-man study. Mol Imag Biol. 2014;16(6):888–95. 10.1007/s11307-014-0754-z 24915934

[10] Roivainen A, Kahkonen E, Luoto P, Borkowski S, Hofmann B, Jambor I, et al. Plasma pharmacokinetics, whole-body distribution, metabolism, and radiation dosimetry of 68Ga bombesin antagonist BAY 86–7548 in healthy men. J Nucl Med. 2013;54(6):867–72. 23564761 10.2967/jnumed.112.114082

[11] Bakker IL, van Tiel ST, Haeck J, Doeswijk GN, de Blois E, Segbers M, et al. In Vivo Stabilized SB3, an attractive GRPR antagonist, for pre- and intra-operative imaging for prostate cancer. Mol Imaging Biol. 2018;20(6):973–83. 29556947 10.1007/s11307-018-1185-z PMC6244536

[12] Guenther T, Deiser S, Felber V, Beck R, Wester HJ. Substitution of L-tryptophan by a-methyl-L-tryptophan in 177Lu-RM2 results in 177Lu-AMTG, a high-affinity gastrin-releasing peptide receptor ligand with improved In vivo stability. J Nucl Med. 2022;63(63):1364–70. 35027371 10.2967/jnumed.121.263323 PMC9454457

[13] Günther T, Felber V, Holzleitner N, Joksch M, Suhrbier T, Schwarzenböck S, et al. In vivo stability of the GRPR antagonist [177Lu]Lu-AMTG in prostate cancer patients: a first step towards improved radioligand therapy. J Nucl Med. 2024;65: 242235.

[14] Kurth J, Heuschkel M, Felber V, Joksch M, Koller L, Wester H-J, et al. GRPr antagonist [177Lu]Lu-AMTG for treatment of castration resistant prostate cancer: first in-human biodistribution and dosimetry. J Nucl Med. 2024;65: 242300.

[15] Shipp MA, Tarr GE, Chen CY, Switzer SN, Hersh LB, Stein H, et al. CD10neutral endopeptidase 2411 hydrolyzes bombesin-like peptides and regulates the growth of small cell carcinomas of the lung. Proc Natl Acad Sci. 1991;88:10662–6. 1660144 10.1073/pnas.88.23.10662 PMC52990

[16] Nock BA, Maina T, Krenning EP, de Jong M. To serve and protect: enzyme inhibitors as radiopeptide escorts promote tumor targeting. J Nucl Med. 2014;55(1):121–7. 24287321 10.2967/jnumed.113.129411

[17] Wang L, Chen C-C, Zhang Z, Kuo H-T, Zhang C, Colpo N, Merkens H, Bénard F, Lin K-S. Synthesis and evaluation of Novel 68Ga-labeled [D-Phe6,Leu13ψThz14]bombesin(6-14) Analogs for cancer imaging with positron emission tomography. Pharmaceuticals. 2024;17(5):621. 10.3390/ph17050621. 38794191 10.3390/ph17050621 PMC11124507

[18] Wang L, Kuo HT, Zhang Z, Zhang C, Chen CC, Chapple D, et al. Unnatural amino acid substitutions to improve in vivo stability and tumor uptake of (68)Ga-labeled GRPR-targeted TacBOMB2 derivatives for cancer imaging with positron emission tomography. EJNMMI Radiopharm Chem. 2024;9(1):8. 38305955 10.1186/s41181-024-00241-7 PMC10837402

[19] Braune A, Oehme L, Freudenberg R, Hofheinz F, van den Hoff J, Kotzerke J, et al. Comparison of image quality and spatial resolution between (18)F, (68)Ga, and (64)Cu phantom measurements using a digital Biograph Vision PET/CT. EJNMMI Phys. 2022;9(1):58. 36064989 10.1186/s40658-022-00487-7 PMC9445107

[20] Liu T, Liu C, Zhang Z, Zhang N, Guo X, Xia L, et al. (64)Cu-PSMA-BCH: a new radiotracer for delayed PET imaging of prostate cancer. Eur J Nucl Med Mol Imaging. 2021;48(13):4508–16. 34170361 10.1007/s00259-021-05426-9

[21] Johnbeck CB, Knigge U, Loft A, Berthelsen AK, Mortensen J, Oturai P, et al. Head-to-head comparison of (64)Cu-DOTATATE and (68)Ga-DOTATOC PET/CT: a prospective study of 59 patients with neuroendocrine tumors. J Nucl Med. 2017;58(3):451–7. 27660147 10.2967/jnumed.116.180430

[22] Boswell CA, Sun X, Niu W, Weisman GR, Wong EH, Rheingold AL, et al. Comparative in vivo stability of copper-64-labeled cross-bridged and conventional tetraazamacrocyclic complexes. J Med Chem. 2004;47(6):1465–74. 14998334 10.1021/jm030383m

[23] Rylova SN, Stoykow C, Del Pozzo L, Abiraj K, Tamma ML, Kiefer Y, et al. The somatostatin receptor 2 antagonist 64Cu-NODAGA-JR11 outperforms 64Cu-DOTA-TATE in a mouse xenograft model. PLoS ONE. 2018;13(4): e0195802. 29668724 10.1371/journal.pone.0195802 PMC5906006

[24] Koller L, Joksch M, Schwarzenböck S, Kurth J, Heuschkel M, Holzleitner N, et al. Preclinical comparison of the (64)Cu- and (68)Ga-Labeled GRPR-targeted compounds RM2 and AMTG, as Well as First-in-Humans [(68)Ga]Ga-AMTG PET/CT. J Nucl Med. 2023;64(10):1654–9. 37934025 10.2967/jnumed.123.265771

[25] Varasteh Z, Mitran B, Rosenstrom U, Velikyan I, Rosestedt M, Lindeberg G, et al. The effect of macrocyclic chelators on the targeting properties of the 68Ga-labeled gastrin releasing peptide receptor antagonist PEG2-RM26. Nucl Med Biol. 2015;42(5):446–54. 25684649 10.1016/j.nucmedbio.2014.12.009

[26] Mitran B, Varasteh Z, Selvaraju RK, Lindeberg G, Sorensen J, Larhed M, et al. Selection of optimal chelator improves the contrast of GRPR imaging using bombesin analogue RM26. Int J Oncol. 2016;48(5):2124–34. 26983776 10.3892/ijo.2016.3429

[27] Fraker PJ, Speck JC Jr. Protein and cell membrane iodinations with a sparingly soluble chloroamide, 1,3,4,6-tetrachloro-3a,6a-diphrenylglycoluril. Biochem Biophys Res Commun. 1978;80(4):849–57. 637870 10.1016/0006-291x(78)91322-0

[28] Weineisen M, Simecek J, Schottelius M, Schwaiger M, Wester HJ. Synthesis and preclinical evaluation of DOTAGA-conjugated PSMA ligands for functional imaging and endoradiotherapy of prostate cancer. EJNMMI Res. 2014;4(1):63. 26116124 10.1186/s13550-014-0063-1 PMC4452638

[29] Linder KE, Metcalfe E, Arunachalam T, Chen J, Eaton SM, Feng W, et al. In Vitro and in Vivo Metabolism of Lu-AMBA and the synthesis and characterization of its metabolites. Bioconjugate Chem. 2009;20(6):1171–8. 10.1021/bc9000189 19480415

[30] Popp I, Del Pozzo L, Waser B, Reubi JC, Meyer PT, Maecke HR, et al. Approaches to improve metabolic stability of a statine-based GRP receptor antagonist. Nucl Med Biol. 2017;45:22–9. 27865999 10.1016/j.nucmedbio.2016.11.004

[31] Mansi R, Wang X, Forrer F, Waser B, Cescato R, Graham K, et al. Development of a potent DOTA-conjugated bombesin antagonist for targeting GRPr-positive tumours. Eur J Nucl Med Mol Imaging. 2011;38(1):97–107. 20717822 10.1007/s00259-010-1596-9

[32] Holzleitner N, Cwojdzinski T, Beck R, Urtz-Urban N, Hillhouse CC, Grundler PV, et al. Preclinical evaluation of gastrin-releasing peptide receptor antagonists labeled with (161)Tb and (177)Lu: a comparative study. J Nucl Med. 2024;65(3):481–4. 38124121 10.2967/jnumed.123.266233 PMC10924159

[33] Wang L, Bratanovic IJ, Zhang Z, Kuo H-T, Merkens H, Zeisler J, Zhang C, Tan R, Bénard F, Lin K-S. 68Ga-labeled [Thz14]Bombesin(7–14) Analogs: promising GRPR-targeting agonist pet tracers with low pancreas uptake. Molecules. 2023;28(4):1977. 10.3390/molecules28041977. 36838968 10.3390/molecules28041977 PMC9962964

[34] Wang L, Zhang Z, Merkens H, Zeisler J, Zhang C, Roxin A, Tan R, Bénard F, Lin K-S. 68Ga-labeled [Leu13ψThz14]Bombesin(7–14) derivatives: promising GRPR-targeting PET tracers with low pancreas uptake. Molecules. 2022;27(12):3777. 10.3390/molecules27123777. 35744904 10.3390/molecules27123777 PMC9230575

[35] Lau J, Rousseau E, Zhang Z, Uribe CF, Kuo HT, Zeisler J, et al. Positron emission tomography imaging of the gastrin-releasing peptide receptor with a novel bombesin analogue. ACS Omega. 2019;4(1):1470–8. 30775647 10.1021/acsomega.8b03293 PMC6372246

[36] Bratanovic IJ, Zhang C, Zhang Z, Kuo HT, Colpo N, Zeisler J, et al. A radiotracer for molecular imaging and therapy of gastrin-releasing peptide receptor-positive prostate cancer. J Nucl Med. 2022;63(3):424–30. 34301778 10.2967/jnumed.120.257758

[37] Günther T, Konrad M, Stopper L, Kunert J-P, Fischer S, Beck R, Casini A, Wester H-J. Optimization of the pharmacokinetic profile of [99mTc]Tc-N4-bombesin derivatives by modification of the pharmacophoric Gln-Trp sequence. Pharmaceuticals. 2022;15(9):1133. 10.3390/ph15091133. 36145354 10.3390/ph15091133 PMC9500665

[38] Rinscheid A, Gable A, Wienand G, Dierks A, Kircher M, Gunther T, et al. Biodistribution and radiation dosimetry of [(99m)Tc]Tc-N4-BTG in patients with biochemical recurrence of prostate cancer. EJNMMI Res. 2024;14(1):42. 38668903 10.1186/s13550-024-01105-6 PMC11052738

