# Supplementary material for: Studies on the impact of modifications at the Gln-Trp site in RM2-based GRPR ligands
Source: EJNMMI Res. 2025 Sep 1;15:114. doi: 10.1186/s13550-025-01241-7 (PMC12401824; doi:10.1186/s13550-025-01241-7)
Supplement: Supplementary file 1 — Supplementary file1 [file 13550_2025_1241_MOESM1_ESM.docx]

**Studies on the Impact of Modifications at the Gln-Trp site in RM2-Based GRPR Ligands**

**- Supplementary Materials -**

Sebastian Fischer^1,#^, Lena Koller^1^, Sandra Dominelli^1^, Roswitha Beck^1^, Hans-Jürgen Wester^1^, and Thomas Günther^1,2,#,^*

^1^ TUM School of Natural Sciences, Department of Chemistry, Chair of Pharmaceutical Radiochemistry, Technical University of Munich, 85748 Garching, Germany

^2^ Molecular Imaging Program at Stanford (MIPS), Department of Radiology, School of Medicine, Stanford University, Stanford, CA 94305, USA

^#^ both authors contributed equally

* Corresponding author: Thomas Günther, thomas.guenther@tum.de, TUM School of Natural Sciences, Department of Chemistry, Chair of Pharmaceutical Radiochemistry, Technical University of Munich, 85748 Garching, Germany

**Corresponding authors:**

Thomas Günther

Phone: +49.89.289.12203

Technical University of Munich,

Chair of Pharmaceutical Radiochemistry,

Walther-Meissner-Str. 3

85748 Garching

GERMANY

Fax: +49.89.289.12204

E-Mail: [thomas.guenther@tum.de](mailto:thomas.guenther@tum.de)

ORCID: <https://orcid.org/0000-0002-7412-0297>

**1. General information**

The Fmoc-(9-fluorenylmethoxycarbonyl-) and all other protected amino acid analogs were purchased from Bachem Inc. (Bubendorf, Switzerland), Merck KGaA (Darmstadt, Germany) or Iris Biotech GmbH (Marktredwitz, Germany). The *H*-Rink amide ChemMatrix^®^ resin (35-100 mesh particle size, 0.4-0.6 mmol/g loading) was purchased from Merck KGaA (Darmstadt, Germany). CheMatech (Dijon, France) delivered the chelators NODAGA(*^t^*Bu)_3_, NOTA-bis(*^t^*Bu) ester, and DOTA(*^t^*Bu)_3_. Peptide syringes were obtained from VWR International GmbH (Bruchsal, Germany).

All necessary solvents and other organic reagents were purchased from either, Alfa Aesar (Karlsruhe, Germany), Merck KGaA (Darmstadt, Germany) or VWR International GmbH (Bruchsal, Germany). Solid-phase synthesis of the peptides was carried out by manual operation using a Scilogex MX-RL-E Analog Rotisserie Tube Rotator (Scilogex, Rocky Hill, CT, USA). H_2_O was used after purification by a Barnstead MicroPure system (Thermo Fisher Scientific Inc., Waltham, MA, USA).

Analytical and preparative reversed-phase high performance liquid chromatography (RP-HPLC) were performed using Shimadzu gradient systems (Shimadzu Deutschland GmbH, Neufahrn, Germany), each equipped with a SPD-20A UV/Vis detector (220 nm, 254 nm). Different gradients of MeCN (0.1% TFA) in H_2_O (0.1% TFA) were used as eluents for all RP-HPLC operations.

For analytical measurements, a Nucleosil 100 C18 (125 × 4.6 mm, 5 μm particle size) column (CS Chromatographie Service GmbH, Langerwehe, Germany) was used at a flow rate of 1 mL/min. Both, specific gradients and the corresponding retention times *t*_R_ as well as the capacity factor *K*’ are cited in the text.

Preparative RP-HPLC purification was done with a Multospher 100 RP 18 (250 × 10 mm, 5 μm particle size) column (CS Chromatographie GmbH, Langerwehe, Germany) at a constant flow rate of 5 mL/min.

Analytical and preparative radio RP-HPLC was performed using a Nucleosil 100 C18 (5 μm, 125 × 4.0 mm) column (CS Chromatographie GmbH, Langerwehe, Germany). Electrospray ionization-mass spectra for characterization of the substances were acquired on an expression^L^ CMS mass spectrometer (Advion Ltd., Harlow, UK).

For radiolabeling, ^177^LuCl_3_ (Molar Activity (*A*_M_) > 3000 GBq/mg, 740 MBq/mL, 0.04 M HCl, ITM Isotope Technologies Munich SE, Garching, Germany) was used. Radioactivity was detected through connection of the outlet of the UV-photometer to an AceMate 925-Scint NaI(Tl) well-type scintillation counter from EG&G Ortec (Oak Ridge, TN, USA). Radioactive samples were measured by a WIZARD^2®^ 2480 Automatic γ-Counter (Perkin Elmer, Waltham, MA, USA) and determination of *IC*_50_ values was carried out using GraphPad Prism 6 (GraphPad Software Inc*.*, San Diego, CA, USA). For radio TLC, a Scan-RAM^TM^ Scanner with Laura^TM^ software (LabLogic Systems Ltd*.*, Broomhill, Sheffield, United Kingdom) was used.

Lyophilization was accomplished using an Alpha 1‑2 LDplus lyophilizer (Martin Christ Gefriertrocknungsanlagen GmbH, Osterode am Harz, Deutschland) combined with a RZ‑2 vacuum pump (Vacuubrand GmbH & Co KG, Olching, Germany).

For in vitro and in vivo studies, the used nutrition mixture Dulbecco’s modified eagle's medium/Ham’s F-12 (DMEM/F-12, v/v = 1/1, with stable glutamine), fetal bovine serum (FBS Superior), phosphate buffered saline (PBS Dulbecco, without Ca^2+^/Mg^2+^), trypsin/EDTA (0.05%/0.02% in PBS without Ca^2+^/Mg^2+^) solution as well as Hank’s balanced salt solution (HBSS, with 0.35 g/L NaHCO_3_ and Ca^2+^/Mg^2+^) were obtained from Biochrom GmbH (Berlin, Germany). Solution of purified products was applied using Tracepur^®^ H_2_O (Merck KGaA, Darmstadt, Germany). Bovine serum albumin (BSA) was purchased from Merck KGaA (Darmstadt, Germany).

Cells were cultured in CELLSTAR^®^ cell culture flasks and seeded in 24-well plates (Greiner Bio-One GmbH, Kremsmünster, Austria) after being counted with a Neubauer hemocytometer (Paul Marienfeld, Lauda-Königshofen, Germany) using Trypan Blue (0.4% in 0.81% NaCl and 0.06% potassium phosphate) solution (Sigma-Aldrich GmbH, Munich, Germany). Cells were handled inside a MSC Advantage laminar flow cabinet and maintained in a Heracell 150i incubator (Thermo Fisher Scientific Inc., Waltham, MA, USA) at 37 °C in a humidified 5% CO_2_ atmosphere.

**2. Execution protocols**

**2.1. General procedures of solid-phase peptide synthesis (SPPS)**

**On-resin peptide formation** **(GP1)**

The respective side-chain protected Fmoc-AA-OH (1.5 eq.) is dissolved in NMP and pre-activated by adding TBTU (1.5 eq.), HOAt (1.5 eq.) and DIPEA (4.5 eq.). After activation for 10 min, the solution is added to resin-bound free amine peptide and shaken for 1.5 h at rt. Subsequently, the resin is washed with NMP (6 × 20 mL/g resin) and after Fmoc deprotection (GP2), the next amino acid is coupled analogously.

**On-resin Fmoc deprotection (GP2)**

The resin-bound Fmoc-peptide is treated with 20% piperidine in NMP (v/v) for 5 min and subsequently for 15 min. Afterwards, the resin is washed with NMP (6 × 20 mL/g resin).

**Conjugation of chelator (GP3)**

The protected chelator DOTA(*^t^*Bu)_3_, NODAGA(tBu)_3_ or NOTA(*^t^*Bu)_2_ (1.5 eq.) is dissolved in NMP and pre-activated by adding TBTU (1.5 eq.), HOAt (1.5 eq.) and DIPEA (4.5 eq.). After activation for 10 min, the solution is added to resin-bound *N*-terminal deprotected peptide (1.0 eq.) and shaken for 3 h at rt. Subsequently, the resin is washed with NMP (3 × 20 mL/g resin) and DCM (3 × 20 mL/g resin).

**Peptide cleavage from the resin with additional deprotection of acid labile protecting groups (GP4)**

The fully protected resin-bound peptide is washed with DCM, afterwards dissolved in a mixture of TFA/TIPS/DCM (v/v/v; 95/2.5/2.5) and shaken for 45 min. The solution is filtered off and the resin is treated in the same way for another 45 min. Both filtrates are combined and concentrated under a stream of nitrogen. After dissolving the residue in MeOH and precipitation in diethyl ether, the liquid is decanted and the remaining solid is dried.

**2.2. Labeling Experiments**

**2.2.1. Non-Radioactive Complexations**

The purified chelator-containing ligand (10^−3^ M in Tracepur^®^ H_2_O, Merck KGaA, Darmstadt, Germany, 1.0 eq.) and either Cu(Oac)_2_, Ga(NO_3_)_3_ or LuCl_3_ (each 20 mM in Tracepur^®^ H_2_O, 2.5 eq.), respectively, were diluted with Tracepur^®^ H_2_O to a final concentration of 10^−4^ M and heated to 90°C for 30 min. After cooling to room temperature, the crude product was obtained and used for *IC*_50_ studies without further purification.

**2.2.2. Radiolabeling**

**^125^I-Labeling**: Briefly, 0.2 mg of [D-Tyr^6^]MJ9 were dissolved in 20 µL Tracepur^®^ H_2_O and 280 µL TRIS buffer (25 mM TRIS ∙ HCl, 0.4 M NaCl, *pH* = 7.9). After solution was transferred to a vial containing 150 µg surface-bound Iodo-Gen^®^ (1,3,4,6-Tetrachloro-3*α*,6*α*-diphenylglycouril, Merck KGaA, Darmstadt, Germany), 5.0 µL (16 MBq) ^125^I-NaI (74 TBq/mmol, 3.1 GBq/mL, 40 mM NaOH, Hartmann Analytic, Braunschweig, Germany) were added. The reaction solution was incubated for 15 min at rt and purified by RP-HPLC. Immediately after purification, sodium ascorbate (0.1 M in Tracepur^®^ H_2_O, 10 vol-%) was added to prevent radiolysis.

RP-HPLC (20→35% MeCN in H_2_O (+ 0.1 TFA) in 20 min): *t_R_* = 18.9 min, *K’* = 10.46.

Structural formula of the radiolabeled reference [3-[^125^I]I-D-Tyr^6^]MJ9.

**^64^Cu-Labeling**: A solution of the purified chelator-containing ligand (10^−3^ M in Tracepur^®^ H_2_O, 1 µL), NaOAc buffer (1.0 M, *pH*= 5.50, 10 µL) and approximately 20 MBq of [^64^Cu]CuCl_2_ (0.08 M in HCl) was heated to 80°C for 10 min. Immediately after labeling, sodium ascorbate (0.1 M, 10 µL) was added to prevent radiolysis. Incorporation of copper-64 was determined by radio-TLC (instant thin layer chromatography paper impregnated with silica gel (iTLC-SG, Agilent Technologies Inc., Folsom, CA, United States); sodium citrate × 1.5 H_2_O (0.1 M)). Radiochemical purity of the labeled compound was determined by radio RP-HPLC.

**^68^Ga-Labeling**: A solution of the purified chelator-containing ligand (10^−3^ M in Tracepur^®^ H_2_O, 1 µL), NaOAc buffer (0.25 M, *pH*= 4.50, 10 µL) and approximately 30 MBq of [^68^Ga]GaCl_3_ (0.04 M in HCl) was heated to 90°C for 10 min. Immediately after labeling, sodium ascorbate (0.1 M, 10 µL) was added to prevent radiolysis. Incorporation of gallium-68 was monitored by radio-TLC (instant thin layer chromatography paper impregnated with silica gel (iTLC-SG, Agilent Technologies Inc., Folsom, CA, United States); sodium citrate × 1.5 H_2_O (0.1 M) + 0.1 M EDTA, *v*/*v* = 9/1). Radiochemical purity of the labeled compound was determined by radio RP-HPLC.

**^177^Lu-Labeling**: A solution of the purified chelator-containing ligand (10^-3^ M in Tracepur^®^ H_2_O, 1 µL), NaOAc buffer (1.0 M, *pH* = 5.50, 10 µL) and approximately 40 MBq of [^177^Lu]LuCl_3_ (0.04 M in HCl) were heated to 95 °C for 10 min. Immediately after labeling, sodium ascorbate (0.1 m, 10 µL) was added to prevent radiolysis. Incorporation of ^177^Lu-lutetium was determined by radio-TLC (instant thin layer chromatography paper impregnated with silica gel (iTLC-SG, Agilent Technologies Inc., Folsom, CA, United States); sodium citrate × 1.5 H_2_O (0.1 M)). Radiochemical purity of the labeled compound was determined by radio-RP-HPLC.

**2.3. *In vitro* experiments**

**2.3.1. Cell Culture**

GRPR-positive PC-3 cells (90112714; Merck KGaA, Darmstadt, Germany) were cultivated in Dulbecco’s modified eagle's medium/Ham’s F-12 (DMEM/F-12, v/v = 1/1, with stable glutamine, Biochrom GmbH, Berlin, Germany) supplemented with fetal bovine serum (10%, FBS Superior, Biochrom GmbH, Berlin, Germany) at 37 °C in a humidified 5% CO_2_ atmosphere. A mixture of trypsin and ethylenediaminetetraacetic acid (0.05%, 0.02%) in PBS (Biochrom GmbH, Berlin, Germany) was used in order to harvest cells. Cells were counted with a Neubauer hemocytometer (Paul Marienfeld, Lauda-Königshofen, Germany).

**2.3.2. Determination of *IC*_50_**

For determination of the GRPR affinity (*IC*_50_), cells were harvested 24 ± 2 h before the experiment and seeded in 24-well plates (1.5 × 10^5^ cells in 1 mL/well).

After removal of the culture medium, the cells were washed once with 500 µL of HBSS (Hank’s balanced salt solution, Biochrom GmbH, Berlin, Germany, with addition of 1% bovine serum albumin (BSA, v/v)) and left in 200 µL HBSS (1% BSA, v/v) for 9 min at rt for equilibration. Next, 25 µL per well of solutions, containing either HBSS (1% BSA, v/v) as control or the respective compound in increasing concentration (10^-10^ M – 10^-4^ M in HBSS (1% BSA, v/v)), were added with subsequent addition of 25 µL of [3-[^125^I]I-D-Tyr^6^]MJ9 (2.0 nm) in HBSS (1% BSA, v/v).

All experiments were performed in triplicate for each concentration. After 2 h incubation at rt, the experiment was terminated by removal of the medium and consecutive rinsing with 300 µL of HBSS (1% BSA, v/v). The media of both steps were combined in one fraction and represent the amount of free radiolabeled reference. Afterwards, the cells were lysed with 300 µL of 1 M NaOH for at least 15 min and united with the 300 µL NaOH of the following washing step. Quantification of bound and free radiolabeled reference was accomplished in a γ-counter. *IC*_50_ determination for each conjugate was repeated twice.

**3. Characterization of RM2-based derivatives**

All mentioned compounds based on the core structure of the golden standard among GRPR antagonists, RM2, were synthesized by standard Fmoc-based SPPS (**GP1**-**4**) using a *H*-Rink amide ChemMatrix^®^ resin (35-100 mesh particle size, 0.4-0.6 mmol/g loading, *Merck KGaA*, Darmstadt, Germany). After finishing the peptide sequence with slightly modifications within the RM2 sequence, a chelator was coupled at the resin (**GP3**). Thereafter, the peptide was cleaved (**GP4**) and purified by RP-HPLC.

**RM2 (DOTA-Pip-D-Phe-Gln-Trp-Ala-Val-Gly-His-Sta-Leu-NH_2_)**

Structural formula of the parent compound RM2.

RP-HPLC (10→90% MeCN in H_2_O (+ 0.1 TFA) in 15 min): *t*_R_ = 6.8 min, *K*‘ = 3.25.

MS (ESI, positive): m/z calculated for C_78_H_118_N_20_O_19_ = 1638.9, found: m/z = 820.2 [M+2H]^2+^, 1639.1 [M+H]^+^.

**Cu-RM2**

RP-HPLC (20→35% MeCN in H_2_O (+ 0.1 TFA) in 20 min): *t*_R_ = 12.9 min, *K*‘ = 7.06.

MS (ESI, positive): m/z calculated for C_78_H_116_CuN_20_O_19_ = 1699.8, found: m/z = 567.8 [M+3H]^3+^, 851.0 [M+2H]^2+^.

**Ga-RM2**

RP-HPLC (20→35% MeCN in H_2_O (+ 0.1 TFA) in 20 min): *t*_R_ = 13.2 min, *K*‘ = 7.25.

MS (ESI, positive): m/z calculated for C_78_H_116_GaN_20_O_19_ = 1705.8, found: m/z = 569.7 [M+3H]^3+^, 854.3 [M+2H]^2+^, 1138.3 [2M+3H]^2+^, 1705.8 [M+H]^+^.

**Lu-RM2**

RP-HPLC (10→90% MeCN in H_2_O (+ 0.1 TFA) in 15 min): *t*_R_ = 6.6 min, *K*‘ = 3.13.

MS (ESI, positive): m/z calculated for C_78_H_115_LuN_20_O_19_ = 1810.8, found: m/z = 906.2 [M+2H]^2+^, 1811.1 [M+H]^+^.

**[Hse^7^]RM2**

Structural formula of [Hse^7^]RM2.

RP-HPLC (10→90% MeCN in H_2_O (+ 0.1 TFA) in 15 min): *t*_R_ = 6.9 min, *K*‘ = 3.31.

MS (ESI, positive): m/z calculated for C_77_H_117_N_19_O_19_ = 1611.9, found: m/z = 807.8 [M+2H]^2+^, 1613.2 [M+H]^+^.

**Cu-[Hse^7^]RM2**

RP-HPLC (10→90% MeCN in H_2_O (+ 0.1 TFA) in 20 min): *t*_R_ = 7.4 min, *K*‘ = 3.10.

MS (ESI, positive): m/z calculated for C_77_H_115_CuN_19_O_19_ = 1672.8, found: m/z = 837.6 [M+2H]^2+^, 558.4 [M+3H]^3+^.

**Ga-[Hse^7^]RM2**

RP-HPLC (10→90% MeCN in H_2_O (+ 0.1 TFA) in 20 min): *t*_R_ = 7.3 min, *K*‘ = 3.10.

MS (ESI, positive): m/z calculated for C_77_H_115_GaN_19_O_19_ = 1678.8, found: m/z = 839.8 [M+2H]^2+^, 560.5 [M+3H]^3+^.

**Lu-[Hse^7^]RM2**

RP-HPLC (10→90% MeCN in H_2_O (+ 0.1 TFA) in 15 min): *t*_R_ = 6.8 min, *K*‘ = 3.25.

MS (ESI, positive): m/z calculated for C_77_H_114_LuN_19_O_19_ = 1783.8, found: m/z = 892.7 [M+2H]^2+^, 1784.5 [M+H]^+^.

**[Bta^8^]****RM2**

Structural formula of [Bta^8^]RM2.

RP-HPLC (10→90% MeCN in H_2_O (+ 0.1 TFA) in 15 min): *t*_R_ = 7.1 min, *K*‘ = 3.44.

MS (ESI, positive): m/z calculated for C_78_H_117_N_19_O_19_S = 1655.9, found: m/z = 829.2 [M+2H]^2+^, 1657.2 [M+H]^+^.

**Cu-[Bta^8^]RM2**

RP-HPLC (10→90% MeCN in H_2_O (+ 0.1 TFA) in 20 min): *t*_R_ = 7.6 min, *K*‘ = 3.22.

MS (ESI, positive): m/z calculated for C_78_H_115_CuN_19_O_19_S = 1716.8, found: m/z = 859.2 [M+2H]^2+^, 573.3 [M+3H]^3+^.

**Ga-[Bta^8^]RM2**

RP-HPLC (10→90% MeCN in H_2_O (+ 0.1 TFA) in 20 min): *t*_R_ = 7.4 min, *K*‘ = 3.11.

MS (ESI, positive): m/z calculated for C_78_H_115_GaN_19_O_19_S = 1722.8, found: m/z = 861.8 [M+2H]^2+^, 574.9 [M+3H]^3+^.

**Lu-[Bta^8^]RM2**

RP-HPLC (10→90% MeCN in H_2_O (+ 0.1 TFA) in 15 min): *t*_R_ = 7.0 min, *K*‘ = 3.38.

MS (ESI, positive): m/z calculated for C_78_H_114_LuN_19_O_19_S = 1827.8, found: m/z = 915.1 [M+2H]^2+^, 1828.9 [M+H]^+^.

**AMTG ([*α*-Me-Trp^8^]RM2)**

Structural formula of [α-Me-Trp^8^]RM2 (AMTG).

RP-HPLC (10→90% MeCN in H_2_O (+ 0.1 TFA) in 15 min): *t*_R_ = 6.9 min, *K*‘ = 3.31.

MS (ESI, positive): m/z calculated for C_79_H_120_N_20_O_19_ = 1652.9, found: m/z = 826.9 [M+2H]^2+^, 1652.6 [M+H]^+^.

**Cu-AMTG**

RP-HPLC (20→35% MeCN in H_2_O (+ 0.1 TFA) in 20 min): *t*_R_ = 14.3 min, *K*‘ = 7.94.

MS (ESI, positive): m/z calculated for C_79_H_118_CuN_20_O_19_ =1713.8, found: m/z = 572.1 [M+3H]^3+^, 857.7 [M+2H]^2+^.

**Ga-AMTG**

RP-HPLC (20→35% MeCN in H_2_O (+ 0.1 TFA) in 20 min): *t*_R_ = 14.5 min, *K*‘ = 8.06.

MS (ESI, positive): m/z calculated for C_79_H_118_GaN_20_O_19_ = 1719.8, found: m/z = 573.9 [M+3H]^3+^, 860.3 [M+2H]^2+^.

**Lu-AMTG**

RP-HPLC (10→90% MeCN in H_2_O (+ 0.1 TFA) in 15 min): *t*_R_ = 7.0 min, *K*‘ = 3.38.

MS (ESI, positive): m/z calculated for C_79_H_117_LuN_20_O_19_ = 1824.8, found: m/z = 913.1 [M+2H]^2+^, 1825.6 [M+H]^+^.

**NOTA-MJ9 (NOTA-[des-DOTA]RM2)**


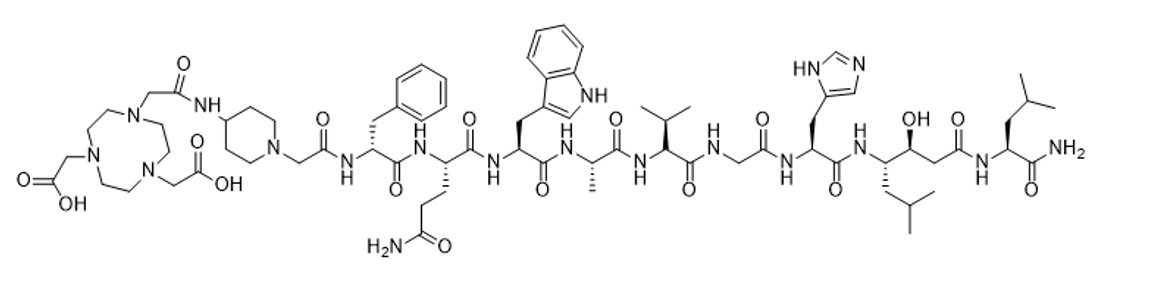


Structural formula of NOTA-MJ9.

MS (ESI, positive): m/z calculated for C_74_H_111_N_19_O_17_ = 1537.8, found: m/z = 769.7 [M+2H]^2+^, 513.5 [M+3H]^3+^.

**Cu-NOTA-MJ9**

RP-HPLC (10→90% MeCN in H_2_O (+ 0.1 TFA) in 15 min): *t*_R_ = 7.2 min, *K*’ = 2.98.

MS (ESI, positive): m/z calculated for C_74_H_109_CuN_19_O_17_ = 1599.4; found: m/z = 800.2 [M+2H]^2+^, 533.8 [M+3H]^3+^.

**Ga-NOTA-MJ9**

RP-HPLC (10→90% MeCN in H_2_O (+ 0.1 TFA) in 15 min): *t*_R_ = 7.1 min, *K*‘ = 2.92.

MS (ESI, positive): m/z calculated for C_74_H_108_GaN_19_O_17_ = 1604.6; found: m/z = 803.6 [M+2H]^2+^, 535.5 [M+3H]^3+^.

**NOTA-[Hse^7^]MJ9 (NOTA-[des-DOTA, Hse^7^]RM2)**


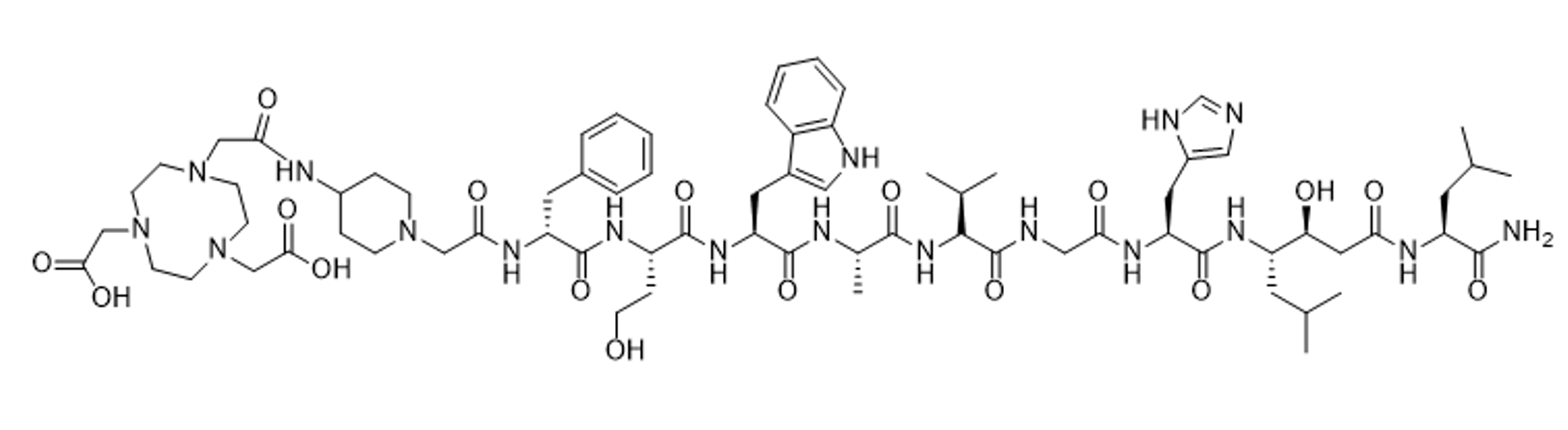


Structural formula of NOTA-[Hse^7^]MJ9.

MS (ESI, positive): m/z calculated for C_73_H_110_N_18_O_17_ = 1510.8; found: m/z = 756.3 [M+2H]^2+^, 504.5 [M+3H]^3+^.

**Cu-NOTA-[Hse^7^]MJ9**

RP-HPLC (10→90% MeCN in H_2_O (+ 0.1 TFA) in 15 min): *t*_R_ = 7.3 min, *K*‘ = 3.04.

MS (ESI, positive): m/z calculated for C_73_H_108_CuN_18_O_17_ = 1572.4; found: m/z = 786.6 [M+2H]^2+^.

**Ga-NOTA-[Hse^7^]MJ9**

RP-HPLC (10→90% MeCN in H_2_O (+ 0.1 TFA) in 15 min): *t*_R_ = 7.2 min, *K*‘ = 2.98.

MS (ESI, positive): m/z calculated for C_73_H_107_GaN_18_O_17_ = 1577.6; found: m/z = 789.4 [M+2H]^2+^, 527.1 [M+3H]^3+^.

**NOTA-[Bta^8^]MJ9 (NOTA-[des-DOTA, Bta^8^]RM2)**


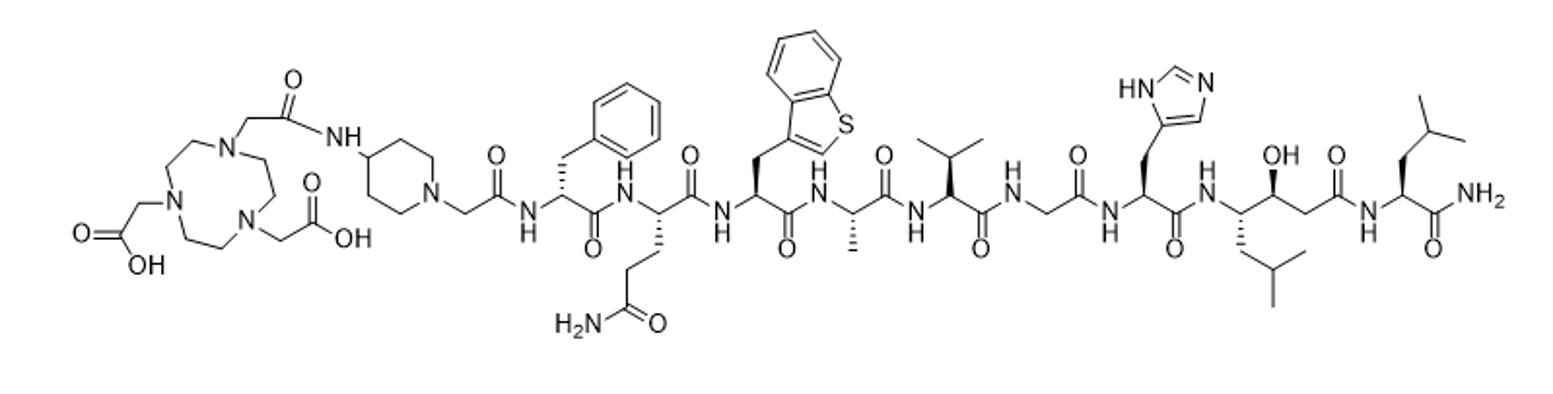


Structural formula of NOTA-[Bta^8^]MJ9.

MS (ESI, positive): m/z calculated for C_74_H_110_N_18_O_17_S = 1554.8; found: m/z = 778.3 [M+2H]^2+^, 519.2 [M+3H]^3+^.

**Cu-NOTA-[Bta^8^]MJ9**

RP-HPLC (10→90% MeCN in H_2_O (+ 0.1 TFA) in 15 min): *t*_R_ = 7.5 min, *K*’ = 3.17.

MS (ESI, positive): m/z calculated for C_74_H_108_CuN_18_O_17_S = 1616.4; found: m/z = 808.6 [M+2H]^2+^, 539.6 [M+3H]^3+^.

**Ga-NOTA-[Bta^8^]MJ9**

RP-HPLC (10→90% MeCN in H_2_O (+ 0.1 TFA) in 15 min): *t*_R_ = 7.4 min, *K*’ = 3.11.

MS (ESI, positive): m/z calculated for C_74_H_107_GaN_18_O_17_S = 1621.5; found: m/z = 811.4 [M+2H]^2+^, 541.0 [M+3H]^3+^.

**NOTA-[α-Me-Trp^8^]MJ9 (NOTA-[des-DOTA, α-Me-Trp^8^]RM2)**


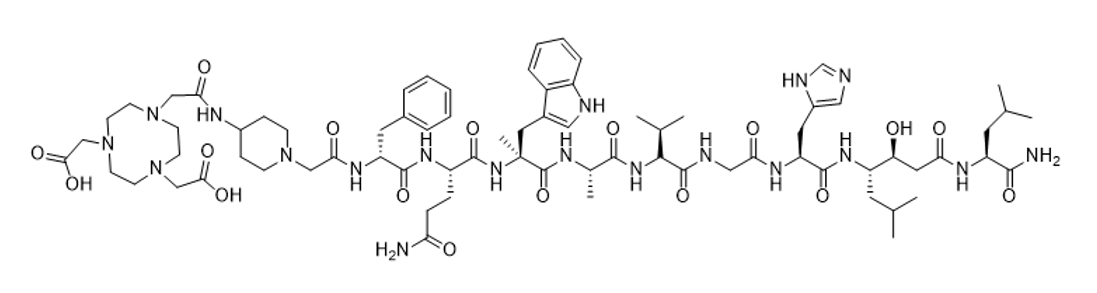


Structural formula of NOTA-[α-Me-Trp^8^]MJ9.

MS (ESI, positive): m/z calculated for C_75_H_113_N_19_O_17_ = 1551.9; found: m/z = 776.7 [M+2H]^2+^, 518.1 [M+3H]^3+^.

**Cu-NOTA-[α-Me-Trp^8^]MJ9**

RP-HPLC (10→90% MeCN in H_2_O (+ 0.1 TFA) in 15 min): *t*_R_ = 7.4 min, *K*’ = 3.10.

MS (ESI, positive): m/z calculated for C_75_H_111_CuN_19_O_17_ = 1612.6; found: m/z = 807.2 [M+2H]^2+^, 539.1 [M+3H]^3+^.

**Ga-NOTA-[α-Me-Trp^8^]MJ9**

RP-HPLC (10→90% MeCN in H_2_O (+ 0.1 TFA) in 15 min): *t*_R_ = 7.3 min, *K*’ = 3.07.

MS (ESI, positive): m/z calculated for C_75_H_110_GaN_19_O_17_ = 1617.7; found: m/z = 809.6 [M+2H]^2+^, 540.0 [M+3H]^3+^.

**NODAGA-MJ9 (NODAGA-[des-DOTA]RM2)**


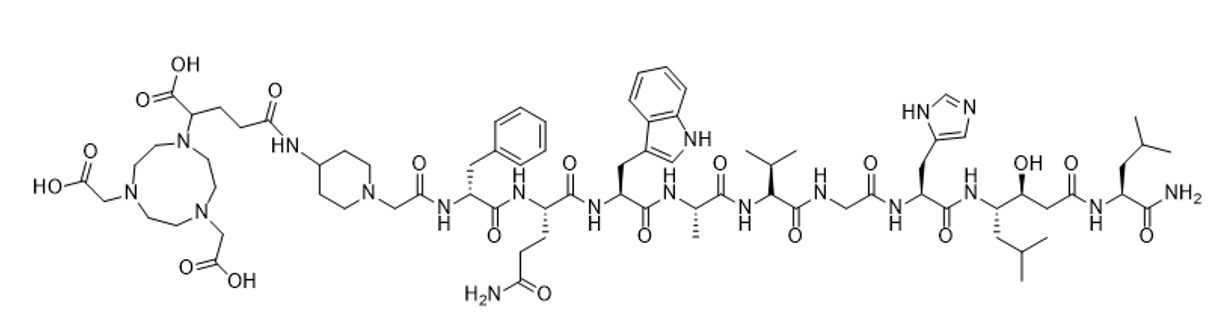


Structural formula of NODAGA-MJ9.

MS (ESI, positive): m/z calculated for C_77_H_115_N_19_O_19_ = 1609.9; found: m/z = 804.9 [M+2H]^2+^, 536.8 [M+3H]^3+^.

**Cu-NODAGA-MJ9**

RP-HPLC (10→90% MeCN in H_2_O (+ 0.1 TFA) in 15 min): *t*_R_ = 7.1 min, *K*‘ = 2.98.

MS (ESI, positive): m/z calculated for C_77_H_113_CuN_19_O_19_ = 1671.4; found: m/z = 836.1 [M+2H]^2+^, 557.8 [M+3H]^3+^.

**Ga-NODAGA-MJ9**

RP-HPLC (10→90% MeCN in H_2_O (+ 0.1 TFA) in 15 min): *t*_R_ = 7.1 min, *K*‘ = 2.97.

MS (ESI, positive): m/z calculated for C_77_H_112_GaN_19_O_19_ = 1676.6; found: m/z = 838.7 [M+2H]^2+^.

**NODAGA-[Hse^7^]MJ9 (NODAGA-[des-DOTA, Hse^7^]RM2)**


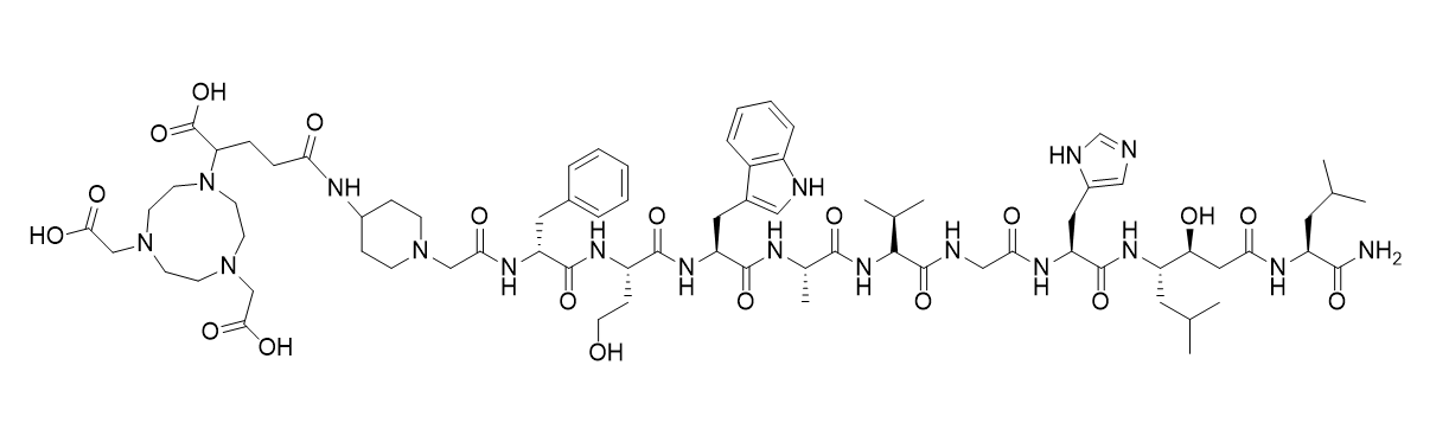


Structural formula of NODAGA-[Hse^7^]MJ9.

MS (ESI, positive): m/z calculated for C_76_H_114_N_18_O_19_ = 1582.9; found: m/z = 792.2 [M+2H]^2+^, 528.5 [M+3H]^3+^.

**Cu-NODAGA-[Hse^7^]MJ9**

RP-HPLC (10→90% MeCN in H_2_O (+ 0.1 TFA) in 15 min): *t*_R_ = 7.3 min, *K*‘ = 3.04.

MS (ESI, positive): m/z calculated for C_76_H_112_CuN_18_O_19_ = 1644.4; found: m/z = 822.4 [M+2H]^2+^.

**Ga-NODAGA-[Hse^7^]MJ9**

RP-HPLC (10→90% MeCN in H_2_O (+ 0.1 TFA) in 15 min): *t*R = 7.3 min, K‘ = 3.06.

MS (ESI, positive): m/z calculated for C_76_H_111_GaN_18_O_19_ = 1649.6; found: m/z = 825.2 [M+2H]^2+^.

**NODAGA-[Bta^8^]MJ9 (NODAGA-[des-DOTA, Bta^8^]RM2)**


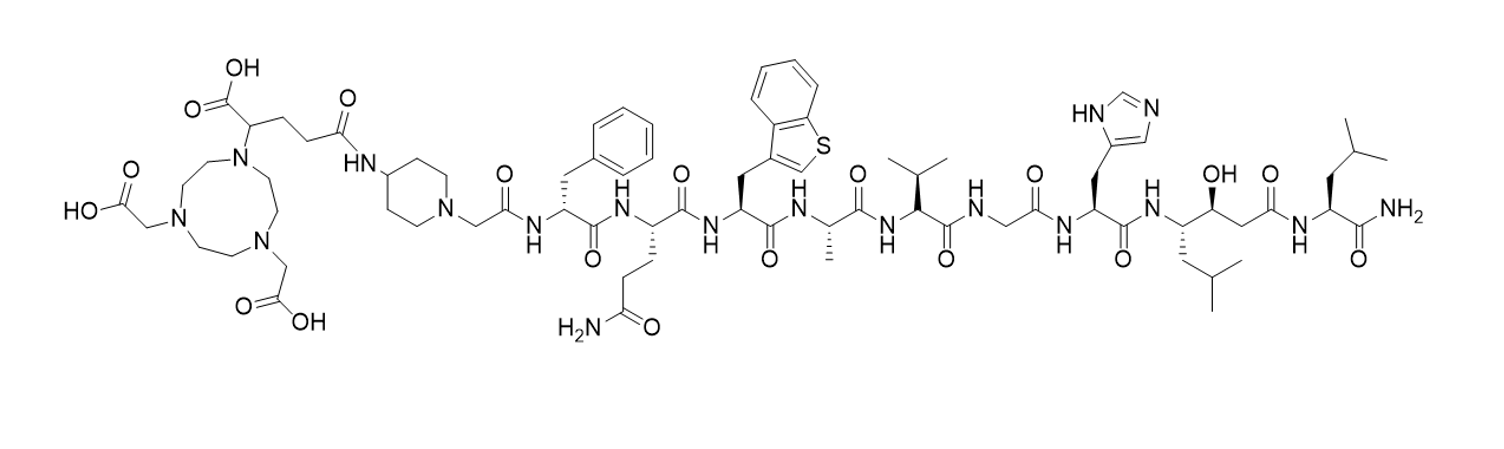


Structural formula of NODAGA-[Bta^8^]MJ9.

MS (ESI, positive): m/z calculated for C_77_H_114_N_18_O_19_S = 1626.8; found: m/z = 813.5 [M+2H]^2+^, 542.6 [M+3H]^3+^.

**Cu-NODAGA-[Bta^8^]MJ9**

RP-HPLC (10→90% MeCN in H_2_O (+ 0.1 TFA) in 15 min): *t*_R_ = 7.5 min, *K*’ = 3.16.

MS (ESI, positive): m/z calculated for C_77_H_112_CuN_18_O_19_S = 1688.4; found: m/z = 844.6 [M+2H]^2+^.

**Ga-NODAGA-[Bta^8^]MJ9**

RP-HPLC (10→90% MeCN in H_2_O (+ 0.1 TFA) in 15 min): *t*_R_ = 7.5 min, *K*’ = 3.18.

MS (ESI, positive): m/z calculated for C_77_H_111_GaN_18_O_19_S = 1693.5; found: m/z = 846.9 [M+2H]^2+^.

**NODAGA-[α-Me-Trp^8^]MJ9 (NODAGA-[des-DOTA, α-Me-Trp^8^]RM2)**


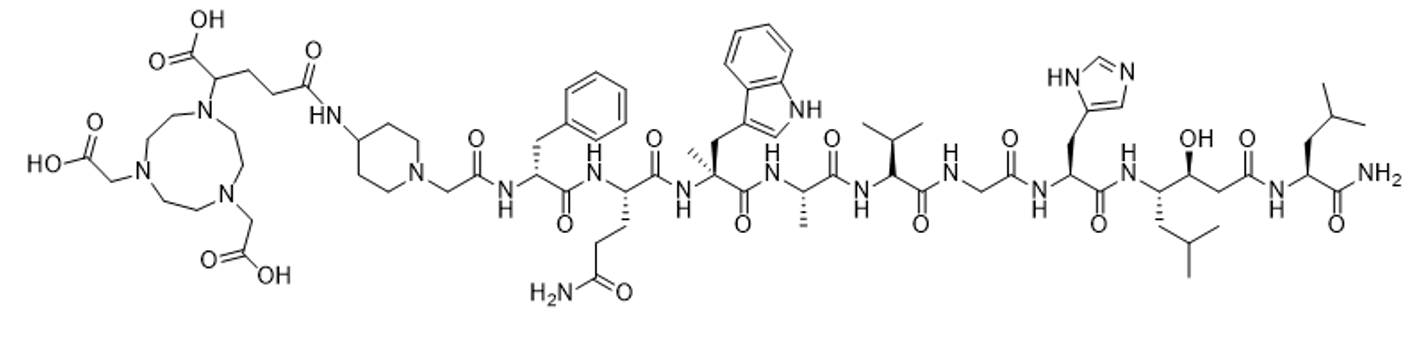


Structural formula of NODAGA-[α-Me-Trp^8^]MJ9.

MS (ESI, positive): m/z calculated for C_78_H_117_N_19_O_19_ = 1623.9; found: m/z = 812.1 [M+2H]^2+^, 541.3 [M+3H]^3+^.

**Cu-NOTA-[α-Me-Trp^8^]MJ9**

RP-HPLC (10→90% MeCN in H_2_O (+ 0.1 TFA) in 15 min): *t*_R_ = 7.4 min, *K*’ = 3.10.

MS (ESI, positive): m/z calculated for C_78_H_114_GaN_19_O_19_ = 1685.4; found: m/z = 843.0 [M+2H]^2+^.

**Ga-NOTA-[α-Me-Trp^8^]MJ9**

RP-HPLC (10→90% MeCN in H_2_O (+ 0.1 TFA) in 15 min): *t*_R_ = 7.3 min, *K*’ = 3.07.

MS (ESI, positive): m/z calculated for C_78_H_114_GaN_19_O_19_ = 1690.6; found: m/z = 845.3 [M+2H]^2+^.

# **4. Supplemental Data**

**Table S1**. Biodistribution of the parent compound [^68^Ga]Ga-RM2 as well as the RM2-based analogs [^68^Ga]Ga-[Hse^7^]RM2, [^68^Ga]Ga-[Bta^8^]RM2, and [^68^Ga]Ga-AMTG in selected organs (in %ID/g) at 1 h p.i. in PC-3 tumor-bearing CB17-SCID mice (100 pmol each). Data is expressed as mean ± SD (n = 4).

| **Organ** | **[^68^Ga]Ga-RM2** | **[^68^Ga]Ga-[Hse^7^]RM2** | **[^68^Ga]Ga-[Bta^8^]RM2** | **[^68^Ga]Ga-AMTG** |
| --- | --- | --- | --- | --- |
| **Blood** | 0.59 ± 0.12 | 3.92 ± 0.07 | 1.07 ± 0.27 | 0.78 ± 0.22 |
| **Heart** | 0.27 ± 0.09 | 1.29 ± 0.14 | 0.40 ± 0.10 | 0.32 ± 0.11 |
| **Lung** | 0.61 ± 0.08 | 2.33 ± 0.28 | 0.92 ± 0.22 | 0.78 ± 0.14 |
| **Liver** | 0.39 ± 0.03 | 1.49 ± 0.06 | 0.73 ± 0.17 | 0.80 ± 0.12 |
| **Spleen** | 0.34 ± 0.05 | 1.55 ± 0.15 | 0.46 ± 0.16 | 0.90 ± 0.73 |
| **Pancreas** | 30.7 ± 2.0 | 8.70 ± 1.12 | 5.19 ± 1.78 | 26.3 ± 6.3 |
| **Stomach** | 3.56 ± 0.42 | 1.88 ± 0.06 | 1.07 ± 0.14 | 3.23 ± 0.37 |
| **Intestine** | 2.67 ± 1.14 | 1.52 ± 0.10 | 0.66 ± 0.14 | 3.64 ± 0.55 |
| **Kidney** | 3.34 ± 0.54 | 3.51 ± 0.20 | 2.33 ± 0.25 | 3.17 ± 0.90 |
| **Adrenal** | 1.10 ± 0.60 | 2.47 ± 0.30 | 1.95 ± 1.30 | 4.56 ± 2.92 |
| **Muscle** | 0.12 ± 0.03 | 0.34 ± 0.04 | 0.19 ± 0.11 | 0.12 ± 0.03 |
| **Bone** | 0.32 ± 0.08 | 0.98 ± 0.12 | 0.30 ± 0.15 | 0.21 ± 0.10 |
| **Tumor** | 14.1 ± 1.9 | 7.62 ± 0.42 | 7.57 ± 1.13 | 15.1 ± 3.7 |

**Table S2**. Tumor-to-background ratios for the selected organs of the parent conjugate [^68^Ga]Ga-RM2 as well as the RM2-based analogs [^68^Ga]Ga-[Hse^7^]RM2, [^68^Ga]Ga-[Bta^8^]RM2, and [^68^Ga]Ga-AMTG at 1 h p.i. in PC-3 tumor-bearing CB17-SCID mice (n = 4). Ratios were determined by the quotient of the mean value of the respective tumor and organ uptake.

| **Organ** | **[^68^Ga]Ga-[Hse^7^]RM2** | **[^68^Ga]Ga-[Bta^8^]RM2** | **[^68^Ga]Ga-AMTG** |
| --- | --- | --- | --- |
| **Blood** | 1.9 ± 0.1 | 7.6 ± 2.6 | 19.6 ± 1.9 |
| **Heart** | 6.0 ± 0.9 | 19.8 ± 5.0 | 50.0 ± 10.6 |
| **Lung** | 3.3 ± 0.6 | 8.8 ± 3.0 | 19.2 ± 1.4 |
| **Liver** | 5.1 ± 0.5 | 10.7 ± 2.3 | 18.8 ± 2.2 |
| **Spleen** | 5.0 ± 0.7 | 18.7 ± 6.9 | 22.8 ± 7.9 |
| **Pancreas** | 0.9 ± 0.1 | 1.9 ± 1.3 | 0.6 ± 0.1 |
| **Stomach** | 4.1 ± 0.3 | 7.1 ± 0.7 | 4.7 ± 1.0 |
| **Intestine** | 5.0 ± 0.3 | 12.2 ± 4.5 | 4.1 ± 0.4 |
| **Kidney** | 2.2 ± 0.2 | 3.3 ± 0.6 | 5.0 ± 1.2 |
| **Adrenal** | 1.4 ± 1.1 | 13.3 ± 17.2 | 5.6 ± 3.8 |
| **Muscle** | 22.8 ± 3.4 | 50.6 ± 19.4 | 139 ± 46 |
| **Bone** | 7.8 ± 0.7 | 29.5 ± 8.2 | 78.1 ± 18.0 |

**Table S3**. Biodistribution of the parent compound [^177^Lu]Lu-RM2 as well as the RM2-based analogs [^177^Lu]Lu-[Hse^7^]RM2, [^177^Lu]Lu-[Bta^8^]RM2, and [^177^Lu]Lu-AMTG in selected organs (in %ID/g) at 1 h p.i. in PC-3 tumor-bearing CB17-SCID mice (100 pmol each). Data is expressed as mean ± SD (n = 4).

| **Organ** | **[^177^Lu]Lu-RM2** | **[^177^Lu]Lu-[Hse^7^]RM2** | **[^177^Lu]Lu-[Bta^8^]RM2** | **[^177^Lu]Lu-AMTG** |
| --- | --- | --- | --- | --- |
| **Blood** | 2.1 ± 0.1 | 1.0 ± 0.2 | 1.6 ± 0.1 | 0.6 ± 0.1 |
| **Heart** | 0.9 ± 0.0 | 0.4 ± 0.1 | 0.7 ± 0.0 | 0.3 ± 0.0 |
| **Lung** | 2.2 ± 0.3 | 0.9 ± 0.2 | 1.8 ± 0.2 | 0.7 ± 0.1 |
| **Liver** | 1.0 ± 0.0 | 0.9 ± 0.1 | 1.1 ± 0.1 | 0.7 ± 0.1 |
| **Spleen** | 0.6 ± 0.0 | 0.6 ± 0.2 | 0. 6 ± 0.1 | 0.4 ± 0.1 |
| **Pancreas** | 15.8 ± 2.9 | 8.0 ± 0.5 | 3.7 ± 1.4 | 24.0 ± 2.3 |
| **Stomach** | 1.9 ± 0.1 | 2.2 ± 0.3 | 1.3 ± 0.3 | 3.3 ± 0.4 |
| **Intestine** | 2.5 ± 0.6 | 1.6 ± 0.2 | 1.0 ± 0.3 | 2.7 ± 0.1 |
| **Kidney** | 3.7 ± 0.0 | 4.8 ± 0.3 | 4.4 ± 0.4 | 4.0 ± 0.3 |
| **Adrenal** | 1.5 ± 0.4 | 1.2 ± 0.5 | 1.1 ± 0.1 | 2.1 ± 0.3 |
| **Muscle** | 0.3 ± 0.1 | 0.2 ± 0.0 | 0.2 ± 0.1 | 0.1 ± 0.0 |
| **Bone** | 1.2 ± 0.3 | 0.2 ± 0.0 | 0.9 ± 0.0 | 0.3 ± 0.1 |
| **Tumor** | 12.5 ± 1.8 | 16.6 ± 1.6 | 12.1 ± 1.8 | 14.2 ± 2.9 |

**Table S4**. Biodistribution of the parent compound [^177^Lu]Lu-RM2 as well as the RM2-based analogs [^177^Lu]Lu-[Hse^7^]RM2, [^177^Lu]Lu-[Bta^8^]RM2, and [^177^Lu]Lu-AMTG in selected organs (in %ID/g) at 24 h p.i. in PC-3 tumor-bearing CB17-SCID mice (100 pmol each). Data is expressed as mean ± SD (n = 4).

| **Organ** | **[^177^Lu]Lu-RM2** | **[^177^Lu]Lu-[Hse^7^]RM2** | **[^177^Lu]Lu-[Bta^8^]RM2** | **[^177^Lu]Lu-AMTG** |
| --- | --- | --- | --- | --- |
| **Blood** | 0.01 ± 0.00 | 0.02 ± 0.00 | 0.04 ± 0.04 | 0.00 ± 0.00 |
| **Heart** | 0.06 ± 0.00 | 0.11 ± 0.03 | 0.09 ± 0.01 | 0.02 ± 0.00 |
| **Lung** | 0.10 ± 0.02 | 0.17 ± 0.02 | 0.22 ± 0.11 | 0.04 ± 0.01 |
| **Liver** | 0.45 ± 0.03 | 0.96 ± 0.15 | 0.77 ± 0.09 | 0.14 ± 0.03 |
| **Spleen** | 0.20 ± 0.02 | 0.36 ± 0.03 | 0.32 ± 0.06 | 0.10 ± 0.02 |
| **Pancreas** | 0.43 ± 0.06 | 0.14 ± 0.01 | 0.17 ± 0.02 | 0.56 ± 0.30 |
| **Stomach** | 0.19 ± 0.06 | 0.26 ± 0.01 | 0.27 ± 0.08 | 0.10 ± 0.03 |
| **Intestine** | 0.22 ± 0.04 | 0.38 ± 0.11 | 0.28 ± 0.07 | 0.20 ± 0.10 |
| **Kidney** | 1.79 ± 0.05 | 1.94 ± 0.28 | 2.07 ± 0.23 | 1.16 ± 0.29 |
| **Adrenal** | 0.80 ± 0.16 | 0.61 ± 0.21 | 0.59 ± 0.33 | 0.46 ± 0.22 |
| **Muscle** | 0.01 ± 0.01 | 0.01 ± 0.00 | 0.02 ± 0.02 | 0.01 ± 0.00 |
| **Bone** | 1.31 ± 0.56 | 2.37 ± 1.13 | 2.58 ± 1.05 | 0.05 ± 0.01 |
| **Tumor** | 8.45 ± 0.19 | 1.49 ± 0.33 | 1.73 ± 0.27 | 11.45 ± 0.43 |

**Table S5**. GRPR affinity (*IC*_50_) of suspected fragments after enzymatic cleavage of RM2 derivatives, determined on PC-3 cells (1.5 × 10^5^ cells/well) and [3-[^125^I]I-D-Tyr^6^]MJ9 (c = 0.2 nM) as radiolabeled reference (2 h, rt, HBSS + 1% BSA, *v*/*v*). Data are expressed as mean ± SD.

| **Fragment of RM2 derivative** | ***IC*_50_ (nM), n = 3** |
| --- | --- |
| **Lu-DOTA-Pip-D-Phe-Gln-Trp-OH** | >10,000 |
| **Lu-DOTA-Pip-D-Phe-Gln-OH** | >10,000 |
| **Lu-DOTA-Pip-D-Phe-Hse-Trp-OH** | >10,000 |
| **Lu-DOTA-Pip-D-Phe-Hse-OH** | >10,000 |
| **Lu-DOTA-Pip-D-Phe-Gln-Bta-OH** | >10,000 |

**Table S6**. Impact of the substitution of Gln^7^ or Trp^8^ within the pharmacophore of NeoB and Universal Ligand (UL, [D-Phe^6^, β-Ala^11^, Phe^13^, Nle^14^]Bn_6-14_) on GRPR affinity (*IC*_50_) and metabolic stability in vitro in human serum. Data are expressed as mean ± SD. Affinities were determined on PC-3 cells (1.5 × 10^5^ cells/well) and [3-[^125^I]I-D-Tyr^6^]MJ9 (c = 0.2 nM) as radiolabeled reference (2 h, rt, HBSS + 1% BSA, *v*/*v*). Metabolic stability in vitro was determined in human serum by incubation at 37 °C for 72 ± 2 h (n = 3). * ^nat^Lu-labeled, ** ^177^Lu-labeled

| **GRPR ligand** | ***IC*_50_ (nM), n = 3*** | **Percentage of intact compound in human serum, n = 3**** |
| --- | --- | --- |
| **NeoB** | 4.2 ± 0.1 | 60.8 ± 1.2% |
| **[Hse^7^]NeoB** | 8.3 ± 0.7 | 57.4 ± 2.1% |
| **[Bta^8^]NeoB** | 13.0 ± 3.0 | 36.2 ± 2.6% |
| **[*α*-Me-Trp^8^]NeoB** | 6.8 ± 1.2 | 70.7 ± 2.8% |
| **UL** | 69.4 ± 3.8 | 6.3 ± 0.6% |
| **[Hse^7^]UL** | 463 ± 211 | 13.8 ± 0.7% |
| **[Bta^8^]UL** | 135 ± 11 | 0.0 ± 0.0% |
| **[*α*-Me-Trp^8^]UL** | 101 ± 11 | 14.3 ± 1.2% |

**
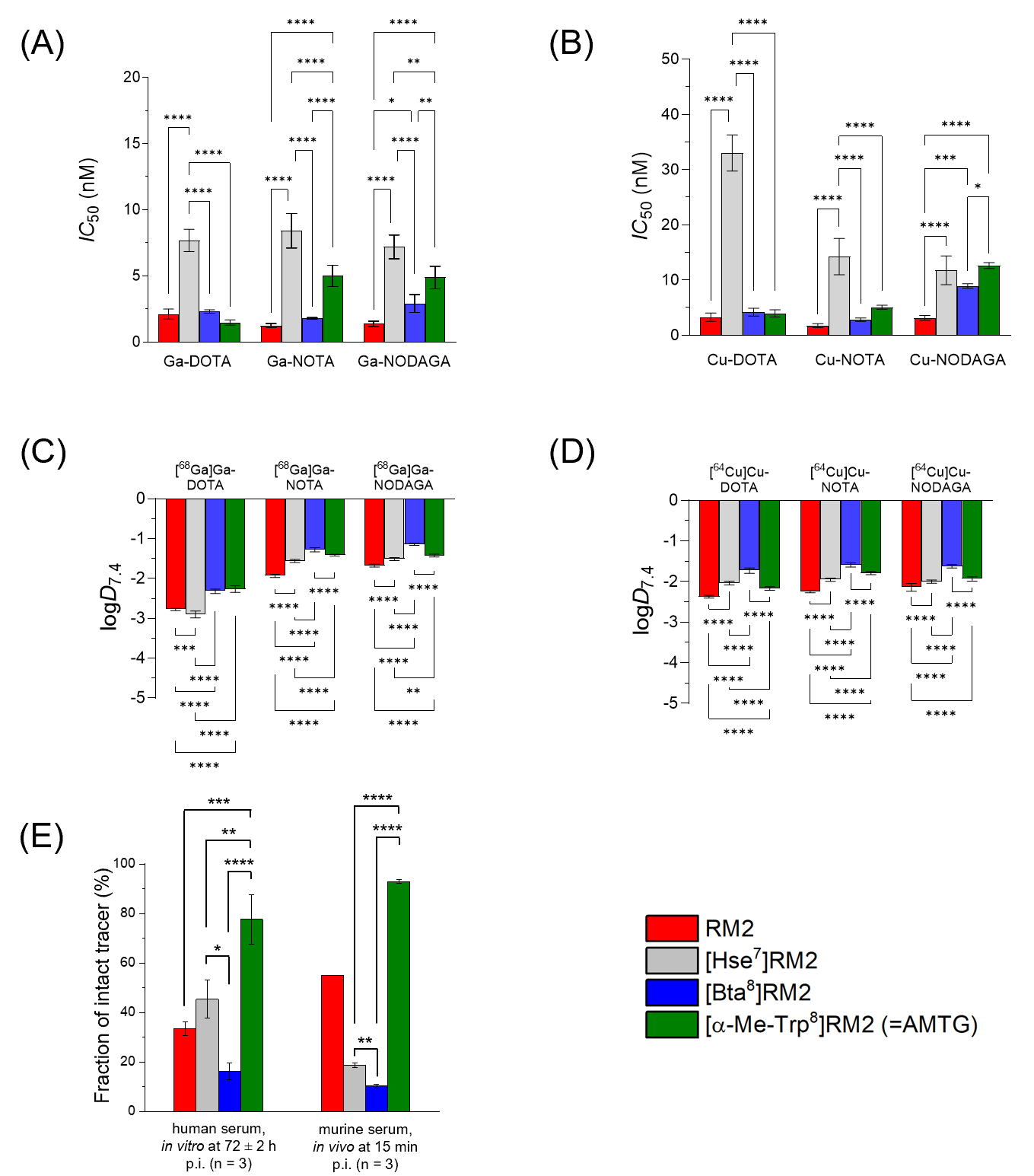
**

**Figure S1.** GRPR affinity (n = 3) and lipophilicity (n = 6) of either ^nat/68^Ga- or ^nat/64^Cu-complexed GRPR ligands containing either a DOTA, NOTA, or NODAGA chelator. *IC*_50_ values of (A) Ga- or (B) Cu-complexed GRPR ligands comprising different chelators. log*D*_7.4_ values of (C) ^68^Ga- or (D) ^64^Cu-complexed GRPR ligands using different chelators. (E) In vitro stability of [^177^Lu]Lu-DOTA-analogs after incubation in human serum at 37 °C for 72 ± 2h (n = 3), and in vivo stability in murine organism at 15 min after injection. * *p* <0.05, ** *p* <0.01, *** *p* <0.001, **** *p* <0.0001; differences that were not statistically significant are not shown in the graph.


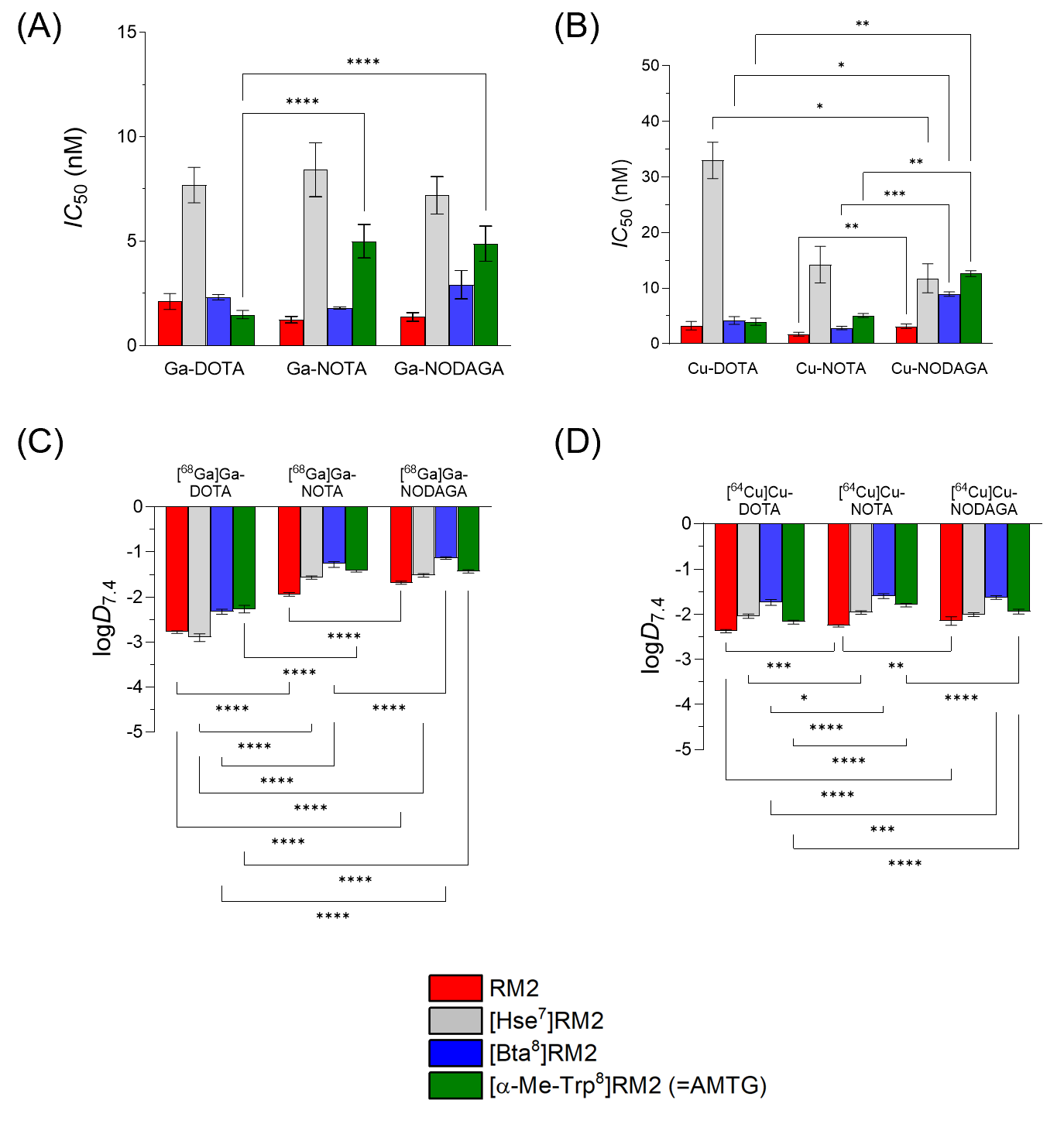


**Figure S2.** GRPR affinity (n = 3) and lipophilicity (n = 6) of either ^nat/68^Ga- or ^nat/64^Cu-complexed GRPR ligands containing either a DOTA, NOTA, or NODAGA chelator. *IC*_50_ values of (A) Ga- or (B) Cu-complexed GRPR ligands comprising different chelators. log*D*_7.4_ values of (C) ^68^Ga- or (D) ^64^Cu-complexed GRPR ligands using different chelators. * *p* <0.05, ** *p* <0.01, *** *p* <0.001, **** *p* <0.0001; differences that were not statistically significant are not shown in the graph


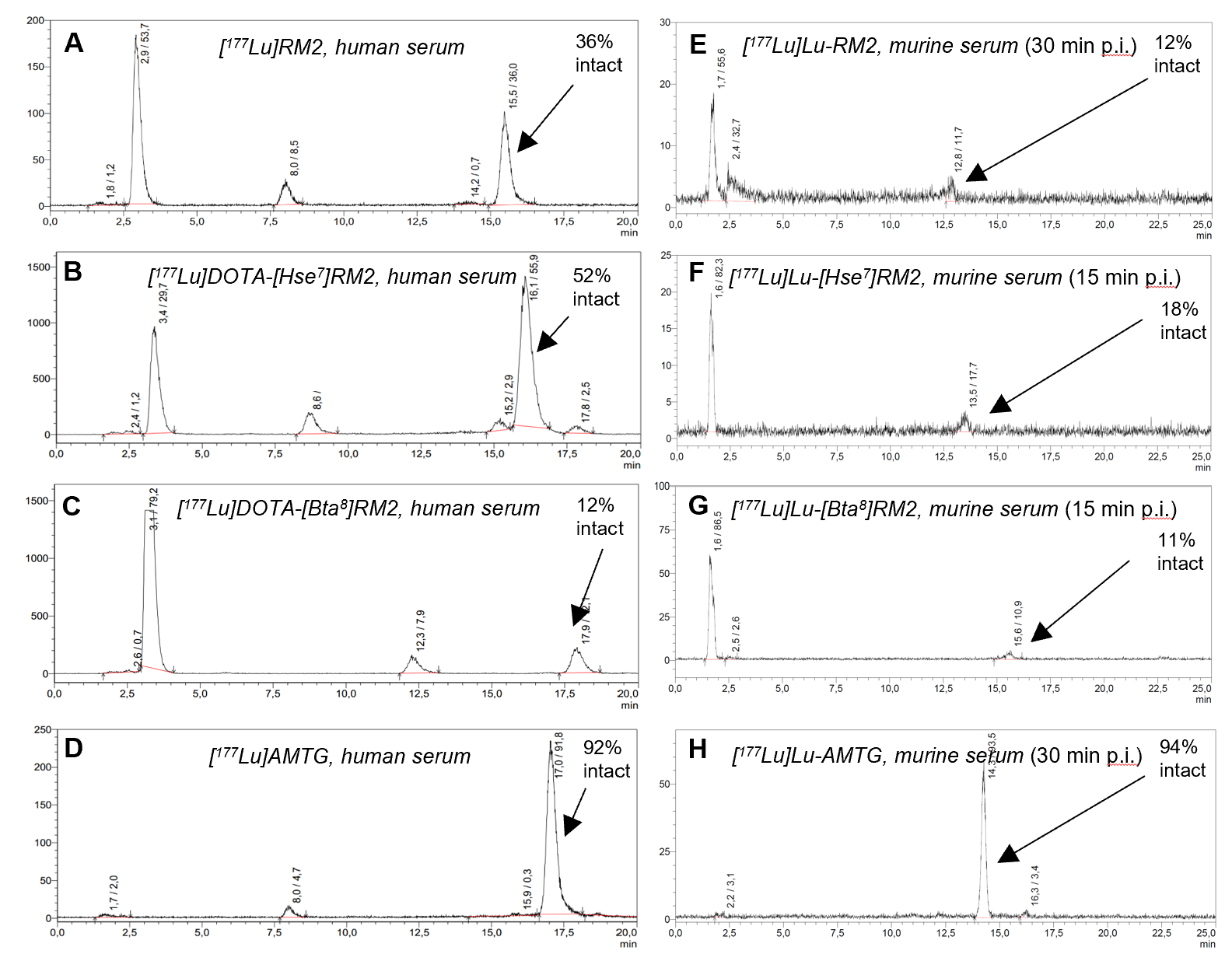


**Figure S3**. Exemplary radio-HPLC chromatograms (20→35% MeCN in H_2_O (+ 0.1% TFA) in 20 min) depicting the stability of ^177^Lu-labeled RM2 analogs either after incubation in vitro in human serum at 37 °C for 72 ± 2 h or in vivo in murine serum (15 min and 30 min after injection, respectively). Stability of (A) [^177^Lu]Lu-RM2 in human serum; (B) [^177^Lu]Lu-[Hse^7^]RM2 in human serum; (C) [^177^Lu]Lu-[Bta^8^]RM2 in human serum; (D) [^177^Lu]Lu-AMTG in human serum; (E) [^177^Lu]Lu-RM2 in murine serum; (F) [^177^Lu]Lu-[Hse^7^]RM2 in murine serum; (G) [^177^Lu]Lu-[Bta^8^]RM2 in murine serum; (H) [^177^Lu]Lu-AMTG in murine serum. Fractions representing the intact, non-metabolized tracers are indicated by black arrows.


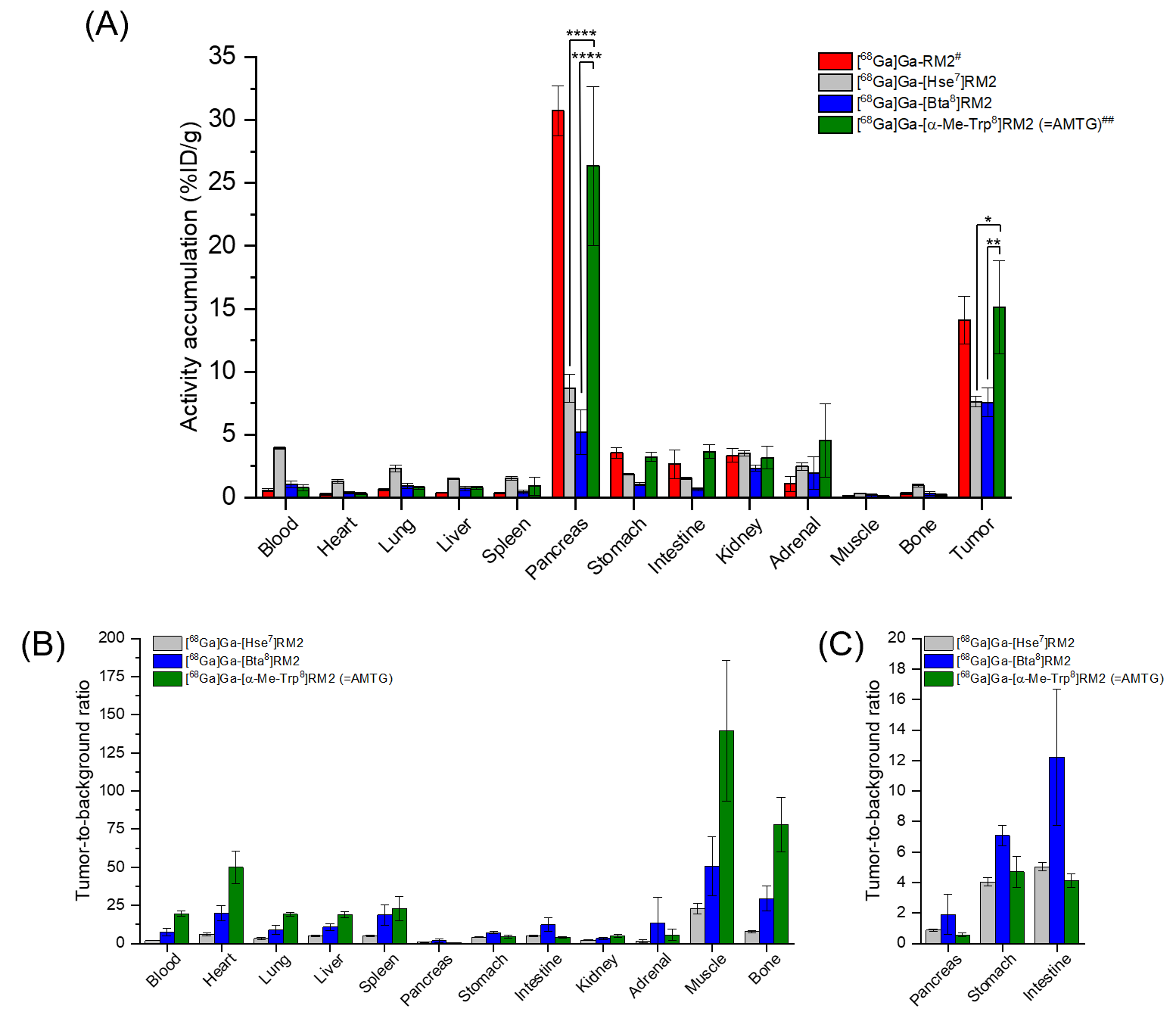


**Figure S4.** In vivo data of ^68^Ga-labeled RM2 derivatives in PC-3 tumor-bearing CB17 SCID mice at 1 h p.i. (n = 4 each, 100 pmol each). Biodistribution (A) and tumor-to-background ratios (B), as well as tumor-to-pancreas, tumor-to-stomach, and tumor-to-intestine ratios (C) of ^68^Ga-labeled GRPR ligands. * p <0.05, ** p <0.01, *** p <0.001, **** p <0.0001; differences that were not statistically significant are not shown in the graph (only pancreas and tumor were compared).


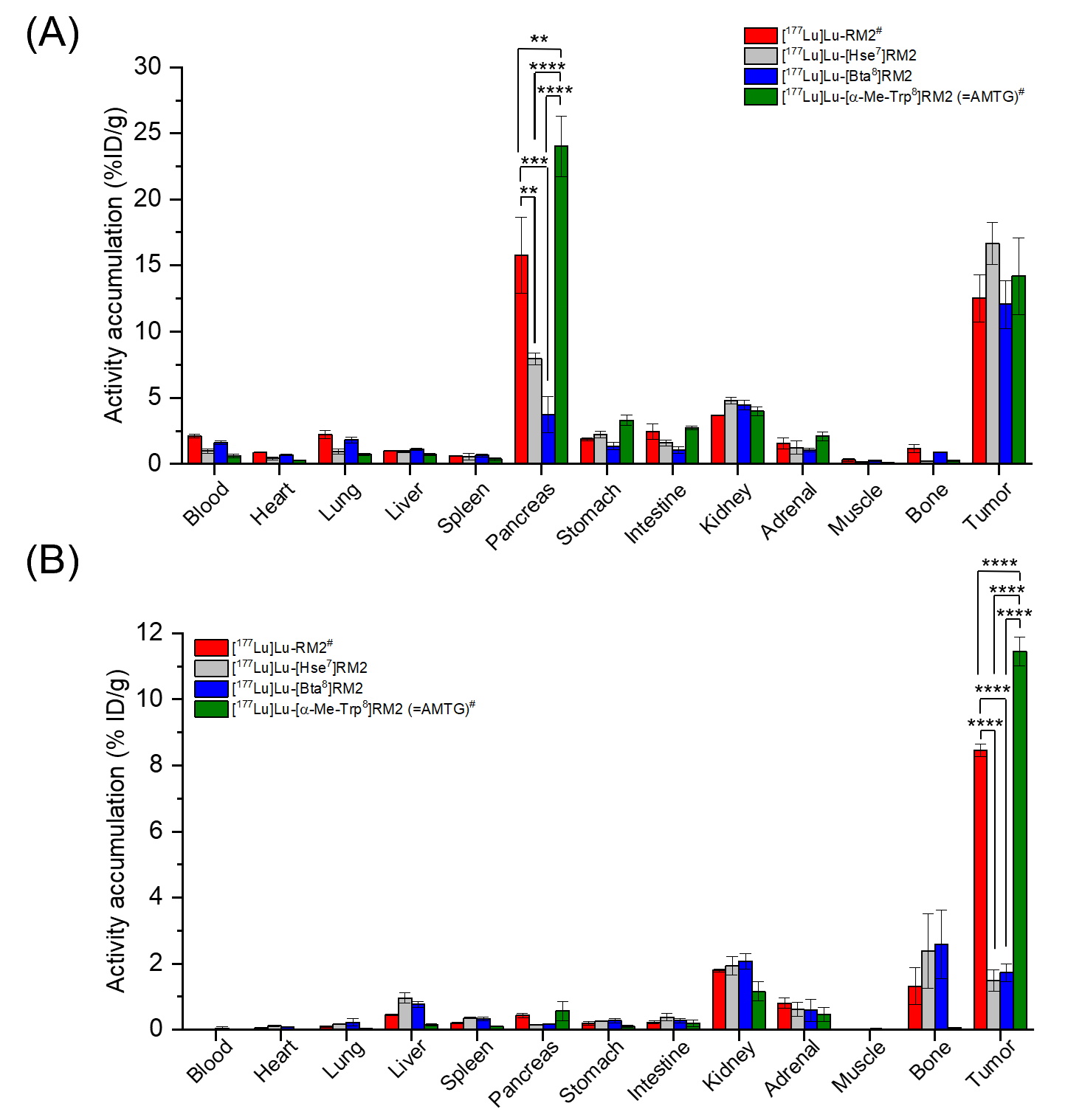


**Figure S5.** In vivo data of ^177^Lu-labeled RM2 derivatives in PC-3 tumor-bearing CB17 SCID mice at (A) 1 h p.i. and (B) 24 h p.i. (n = 4 each, 100 pmol each). ** p <0.01, *** p <0.001, **** p <0.0001; differences that were not statistically significant are not shown in the graph (only pancreas and tumor were compared).


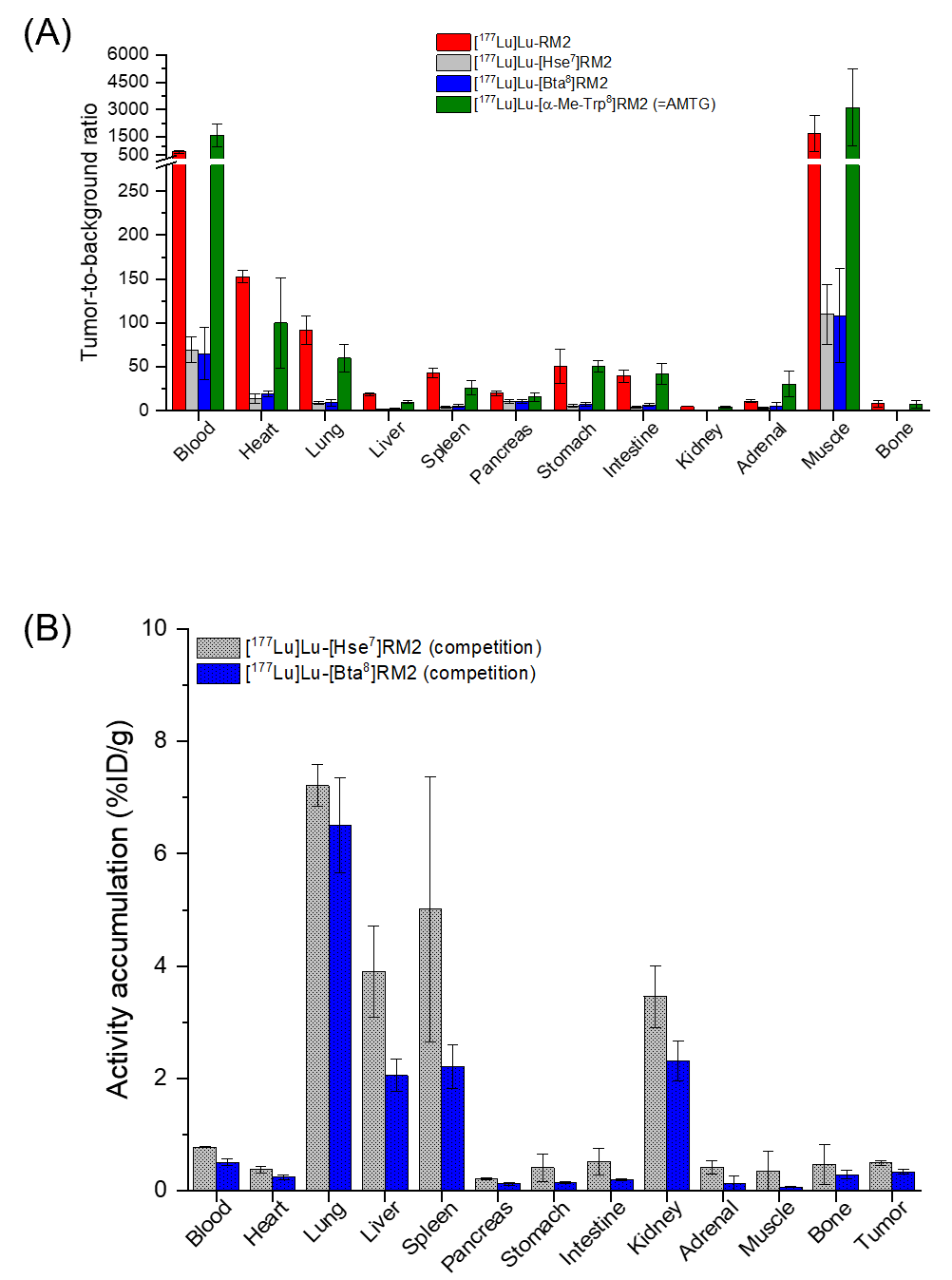


**Figure S6**. (A) Tumor-to-background ratios for the selected organs of ^177^Lu-labeled RM2 analogs at 24 h p.i. in PC-3 tumor-bearing CB17-SCID mice. Data are expressed as mean ± SD (n = 4). (B) Biodistribution data of [^177^Lu]Lu-[Hse^7^]RM2 and [^177^Lu]Lu-[Bta^8^]RM2 (100 pmol, 1 h after injection) in PC-3 tumor-bearing CB17-SCID mice co-injected with 3.62 mg/kg (40 nmol) of Lu-RM2 (10^−3^ M in PBS). Data are expressed as mean ± SD (n = 3).
